# Temporal trends and predictors of antimicrobial resistance among *Staphylococcus* spp. isolated from canine specimens submitted to a diagnostic laboratory

**DOI:** 10.1371/journal.pone.0200719

**Published:** 2018-08-01

**Authors:** Julia G. Conner, Jackie Smith, Erdal Erol, Stephan Locke, Erica Phillips, Craig N. Carter, Agricola Odoi

**Affiliations:** 1 Biomedical and Diagnostic Sciences, College of Veterinary Medicine, University of Tennessee, Knoxville, Tennessee, United States of America; 2 Veterinary Diagnostic Laboratory, University of Kentucky, Lexington, Kentucky, United States of America; Universitatsklinikum Munster, GERMANY

## Abstract

**Background:**

Resistance to commonly used antimicrobials is a growing concern in both human and veterinary medicine. Understanding the temporal changes in the burden of the problem and identifying its determinants is important for guiding control efforts. Therefore, the objective of this study was to investigate temporal patterns and predictors of antimicrobial resistance among *Staphylococcus* spp. isolated from canine specimens submitted to the University of Kentucky Veterinary Diagnostic Laboratory (UKVDL) between 1993 and 2009.

**Methods:**

Retrospective data of 4,972 *Staphylococcus* isolates assessed for antimicrobial susceptibility using the disk diffusion method at the UKVDL between 1993 and 2009 were included in the study. Temporal trends were assessed for each antimicrobial using the Cochran-Armitage trend test. Logistic regression models were used to investigate predictors of antimicrobial resistance (AMR) and multidrug resistance (MDR).

**Results:**

A total of 68.2% (3,388/4,972) *Staphylococcus* isolates were *S*. *intermedius* group (SIG), 18.2% (907/4,972) were coagulase-negative *staphylococci* (CoNS), 7.6% (375/4,972) were *S*. *aureus*, 5.8% (290/4,972) were *S*. *hyicus*, and *S*. *schleiferi* subsp. *coagulans* comprised 0.2% (12/4,972) of the isolates. The overall percentage of AMR and MDR were 77.2% and 25.6%, respectively. The highest levels of AMR were seen in CoNS (81.3%; 737/907), *S*. *aureus* (80.5%; 302/375), and SIG (77.6%; 2,629/3388). The lowest levels of AMR were observed in *S*. *hyicus* (57.9%; 168/290) and *S*. *schleiferi* subsp. *coagulans* (33.3%; 4/12). Overall, AMR and MDR showed significant (p<0.001) decreasing temporal trends. Significant temporal trends (both increasing and decreasing) were observed among 12 of the 16 antimicrobials covering 6 of the 9 drug classes assessed. Thus, significant increasing temporal trends in resistance were observed to β-lactams (p<0.001) (oxacillin, amoxicillin-clavulanate, cephalothin, and penicillin (p = 0.024)), aminoglycosides (p<0.001) (gentamicin, and neomycin), bacitracin (p<0.001), and enrofloxacin (p<0.001). In contrast, sulfonamide (p<0.001) (sulfadiazin) and tetracycline (p = 0.010) resistant isolates showed significant decreasing temporal trends in AMR. *Staphylococcus* spp., geographic region, and specimen source were significant predictors of both AMR and MDR.

**Conclusions:**

Although not unexpected nor alarming, the high levels of AMR to a number of antimicrobial agents and the increasing temporal trends are concerning. Therefore, continued monitoring of AMR among *Staphylococcus* spp. is warranted. Future studies will need to identify local factors responsible for the observed geographic differences in risk of both AMR and MDR.

## Introduction

*Staphylococcus* spp. encompass a diverse group of Gram-positive, non-motile facultative anaerobic cocci that are classified into 3 categories based on production of coagulase: coagulase-positive (CoPS), coagulase-negative (CoNS), and coagulase-variable [1]. *S*. *hyicus*, for instance, is a coagulase-variable species whereas *S*. *aureus* and *S*. *intermedius* group (SIG), which includes *S*. *pseudintermedius*, are coagulase-positive [2–5]. *S*. *pseudintermedius*, the primary staphylococcal pathogen of dogs, is an opportunistic pathogen routinely found on the skin and mucosal surfaces of dogs [2,6,7]. The non-coagulase producing *Staphylococcus* include numerous species such as *S*. *epidermidis* and *S*. *haemolyticus* and are thought to be less or non-pathogenic commensals [1]. However, there is some debate among researchers on the pathogenicity of CoNS, with some studies suggesting that CoNS may play a role in canine dermatitis [8] and nosocomial infections in humans [1].

Resistance, especially acquired multi-drug resistance of CoPS, to commonly used antimicrobials is a growing concern in both human and animal medicine [9,10]. Methicillin‐resistant *S*. *pseudintermedius* (MRSP) infections, in particular, are of growing concern in small animal medicine [11] as they have been reported to play a significant role in skin and surgical site infections [11,12] and lead to significant treatment challenges [6]. Moreover, dogs represent a potential source of methicillin resistant *Staphylococcus aureus* (MRSA) infections or re-infections for humans [13,14] In fact, there is evidence of transfer of resistant organisms between animals and people [15] implying that dogs are of significant public health importance because of their close companionship with people. In the United States, for instance, up to 36.5% (43 million) of households own a dog [16].

Use of antimicrobials is one of the contributing factors to the development of antimicrobial resistance [17] and some authors have suggested that over prescription of antimicrobials may be responsible for the increasing levels of antimicrobial resistance over time [8,18]. Unfortunately, regulatory oversight of antimicrobial use in animals in the United States has focused mainly on food animal production systems with little attention given to their use in companion animals [19]. Most of what is known regarding use of antimicrobials in dogs have been from studies of limited populations. For example, a study by Baker and colleagues, evaluated antimicrobial usage in 435 dogs admitted to a veterinary teaching hospital and found that 55.6% of the dogs had received at least 1 antimicrobial treatment in the previous 12 months while 39.4% had received ≥ 2 antimicrobial treatments [20]. The study also reported that 72.7% of the dogs received β-lactams (cephalexin), 32.2% received aminoglycosides (neomycin and gentamicin), and 23.1% received a fluoroquinolone (enrofloxacin) [20].

Understanding not only the usage patterns of antimicrobials in dogs but also the patterns of antimicrobial resistance and temporal changes is critical for guiding efforts to curb the problem. High levels of antimicrobial resistance to at least one antimicrobial among clinical cases of canine *Staphylococcus* infections have been reported in a number of geographical locations: 88% in Poland [8], 90.9% in Canada [21], and 80.5% in South Africa [22]. Of greater concern are reports of multidrug resistance among *Staphylococcus* isolates in both healthy and clinical cases: 24.5% in Switzerland [23] to 28.7% in South Africa [22] and 34% in the UK [24]. High levels of *Staphylococcus* spp. resistance to β-lactam antimicrobials and lincosamides have been reported by a number of studies [22,25–27] implying that these drugs can no longer be used in the treatment of *Staphylococcus* infections in the concerned geographic areas. With respect to the temporal changes in levels of antimicrobial resistance, the findings are less clear. Some studies have reported no significant temporal changes, others have reported significant increases while others have reported decreasing temporal trends. For instance, a Canadian study by Prescott *et al* [28] reported no significant temporal changes of *S*. *aureus* resistance to fluoroquinolones in dogs treated for urinary tract infections at a veterinary teaching hospital. In contrast, increasing temporal trends in resistance to trimethoprim-sulphamethoxazole among *S*. *pseudintermedius* isolates were reported in a study of healthy and clinical canine pyoderma cases in France [18]. While, a South African study of dogs treated at a veterinary teaching hospital [22] found both significant increasing temporal trends (e.g. enrofloxacin, trimethoprim-sulphamethoxazole, and clindamycin) and significant decreasing temporal trends (e.g. doxycycline, kanamycin, and amoxicillin) in levels of antimicrobial resistance.

It is important to understand not only the burden of antimicrobial resistance but also predictors and temporal changes in resistance to specific drugs and drug classes to better guide treatment decisions as well as efforts to address the problem. Therefore, the objective of this study was to investigate temporal patterns and predictors of antimicrobial resistance among *Staphylococcus* spp. isolated from dog specimens submitted to the University of Kentucky Veterinary Diagnostic Laboratory between 1993 and 2009.

## Methods

### Ethics approval

This study was approved by the University of Tennessee Institutional Animal Care & Use Committee (IACUC). The study used retrospective laboratory records and did not involve animals. All data were handled in compliance with relevant guidelines. No field studies or experiments were conducted as part of this study and hence no informed consent was required.

### Data source

Laboratory records of 4,972 dog specimens submitted to the University of Kentucky Veterinary Diagnostic Laboratory (UKVDL) between January 1993 and July 2009 were included in the study. The records included antimicrobial sensitivity test results, animal demographic information, and geographic information of specimen origin. The following variables were extracted for each case: submission date, accession number, name, city, county, state, zip code, breed, sex, and age of the dog as well as specimen source and *Staphylococcus* species isolated. The criteria used for reporting a microorganism was the isolation of the microorganism in pure culture or significant numbers from specimens (as the predominate microorganism). No duplicate specimens from a single patient were identified. For the isolation of bacteria, specimens were cultured on a Tryptic Soy Agar (TSA) base with 5% horse blood agar and eosin methylene blue agar plates at 37°C in 5–10% CO_2_, for a minimum of 24 hours. If the specimen was from a likely contaminated site such as nasal swab, a Columbia colistin and nalidixic acid (CNA) plate with blood was also inoculated. The CNA plates containing colistin (10 mg/L) and nalidixic acid (10 mg/L) only inhibit gram negative bacteria and therefore should not influence resistance patterns of *Staphylococcus* spp (which is a gram positive organism). The plates were examined for pathogenic bacteria and were incubated for an additional 24 hours at 37°C in aerobic incubators and examined again for pathogenic bacteria. *Staphylococcus* isolates were identified by using colony morphology, dark-field examination, β-hemolysis on the blood agar and CNA plates, and conventional biochemical tests, including coagulase, maltose, mannitol, and trehalose (Table 1). Additionally, selective and differential plates with antibiotics and indicator were used to differentiate between *S*. *aureus* and *S*. *hyicus*.

**Table 1:** The testing scheme used for differentiation of veterinary pathogenic *Staphyloccoccus* spp.

Organism	Biochemical Reactions			
	Coagulase	Maltose	Mannitol	Trehalose
*S*. *aureus*	+	+	+	+
*S*. *schleiferi ss coagulans*	+	-	+	variable
*S*. *lutrae*	+	+	variable	+
*S*. *intermedius*	+	weak	variable	+
*S*. hyicus ss hyicus*	+/-	-	-	+
*S*. delphini*	+	+	+	(+)
*Staphyloccous coagulase negative*	-	-	variable	variable

*Interpretation note: If Staphylococcus isolate is negative for tube coagulase test, it is reported as “coagulase negative *Staphylococcus* species”. Any clot (soft clot) is considered a positive reaction.

Five *Staphylococcus* groups were identified: Coagulase-negative staphylococci (CoNS), *S*. *aureus*, *S*. *hyicus*, *S*. *intermedius* group (SIG), and *S*. *schleiferi* subsp. *coagulans* [29–31]. The laboratory did not specify coagulase negative species or differentiate between *S*. *intermedius* and *S*. *pseudintermedius*. However, since *S*. *pseudintermedius* is the most common *Staphylococcus* spp. of dogs, the majority of SIG isolates are likely *S*. *pseudintermedius*.

For antimicrobial susceptibility testing, *Staphylococcus* isolates were subjected to a panel of 16 drugs using the Kirby-Bauer disc diffusion test. The laboratory followed testing procedures and classification criteria that were in use during the testing period by the Clinical and Laboratory Standards Institute (CLSI) formerly called the National Committee for Clinical Laboratory Standards (NCCLS) [29,32–35] to determine the susceptibility of the isolates. Sizes of zones of susceptible and resistant isolates, in millimeters, were as follows: amoxicillin-clavulanic acid (≥20, ≤19), bacitracin (≥13, ≤8), cephalothin (≥18, ≤14), enrofloxacin (≥21, ≤17), erythromycin (≥21, ≤15), gentamicin (≥15, ≤12), kanamycin (≥18, ≤13), lincomycin (≥19, ≤15), neomycin (≥17, ≤12), novobiocin (≥17, ≤14), oxacillin (≥13, ≤10), penicillin (≥28, ≤19), streptomycin (≥15, ≤11), sulfadiazine (≥17, ≤12), sulfamethoxazole-trimethoprim (≥16, ≤10), and tetracycline (≥23, ≤18). Isolates were classified as susceptible, moderately susceptible, intermediate, or resistant based on the above classification procedure [29,32–35]. The World Health Organization and National Committee for Clinical Laboratory Standards defined “moderately susceptible" isolates as those that can be treated using a higher dosage of the antimicrobial in question whereas those listed as “intermediate” should not be dosed at higher levels due to toxicity concerns [34,36].

### Data preparation

**Fig 1:**
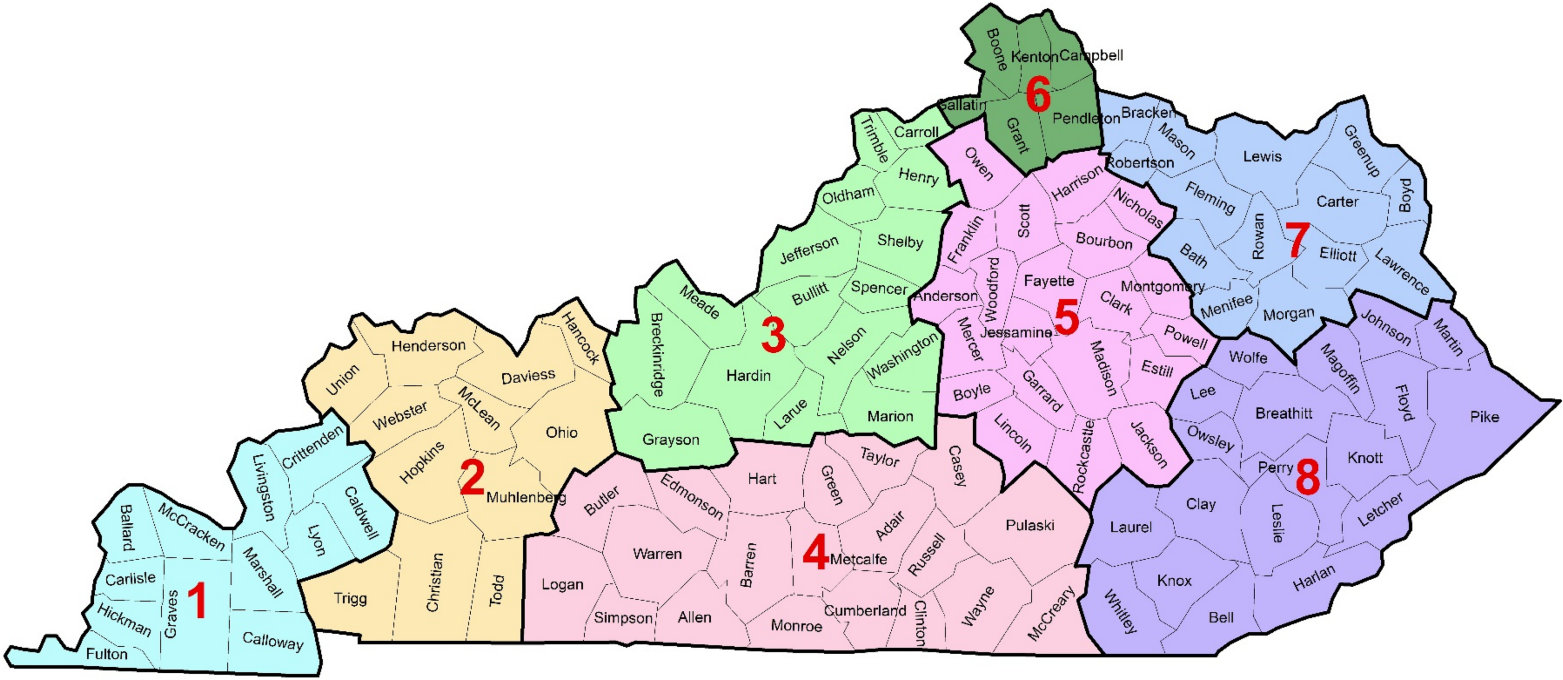
Kentucky regions, based on Centers for Medicare and Medicaid Rating Areas [39], investigated for differences in antimicrobial resistance among canine specimens submitted to the University of Kentucky Veterinary Diagnostic Laboratory.

Data cleaning and preparation were performed in Matlab [37] and Microsoft Excel [38]. Counties were assigned to eight (8) regions based on the Centers for Medicare and Medicaid Services (CMS) rating areas [39] (Fig 1). These regions are classified based on Metropolitan (core urban area of 50,000 or more) and Micropolitan (urban area of 10,000 but less than 50,000) Statistical Areas (MSAs) plus surrounding counties that were determined to have socioeconomic integration to the MSA [39,40]. Region 8 had the highest percentage (29%) of the population living below the poverty level in 1999 (based on 2000 decennial census) and region 6 had the lowest (9%). The percentages of the population living below poverty level for regions 1, 2, 3, 4, 5 and 7 were 15%, 15%, 12%, 19% 14% and 20%, respectively. Cases were assigned to one of the eight regions based on their counties of origin.

Dog breeds were re-coded into groups based on the American Kennel Club (AKC) group classification [41]. Mixed breeds were separated into a non-AKC group designated as mixed (n = 900). Age was categorized into 5 categories: < 2 years, 2–4 years, 4–6 years, 6–8 years, and > 8 years. Sex was defined into 2 categories male and female. In situations where sex was listed by sterilization status (i.e. spayed, neutered, or castrated), it was placed in the appropriate sex category. Specimen source was classified into the following 5 categories: (1) ears, (2) skin, hair, and nails, (3) urine and bladder, (4) mucosal surfaces and (5) “all others”. The “all others” category included non-specific specimen submissions and those with small specimen sizes. Mucosal surfaces included nasal, oral, conjunctival, and vaginal swabs. Antimicrobial susceptibility test results were re-classified as susceptible or resistant. Those listed as moderately susceptible (n = 2,666) or intermediate (n = 1) were re-coded as resistant. Antimicrobials were further classified into their respective drug classes. Two variables were created to identify antimicrobial resistance status: (1) antimicrobial resistance (AMR) defined as resistance to at least one antimicrobial; and (2) multidrug resistance (MDR) defined as resistance to at least one antimicrobial in 3 or more antimicrobial classes [42]. Extensive drug resistance (XDR) was defined as drug resistance to at least one antimicrobial in all but one or two classes [42].

### Statistical analysis

All statistical analyses were performed using IBM SPSS Statistics 24 [43]. Crude and factor-specific percentages of AMR and MDR isolates were computed. The factors considered (suspected categorical predictors of AMR and MDR) were year, *Staphylococcus* spp., geographic region, dog breed, age group, sex, and specimen source. Cochran-Armitage trend test was used to assess temporal trends in AMR and MDR. Statistical significance was assessed using an α of 0.05.

**Fig 2:**
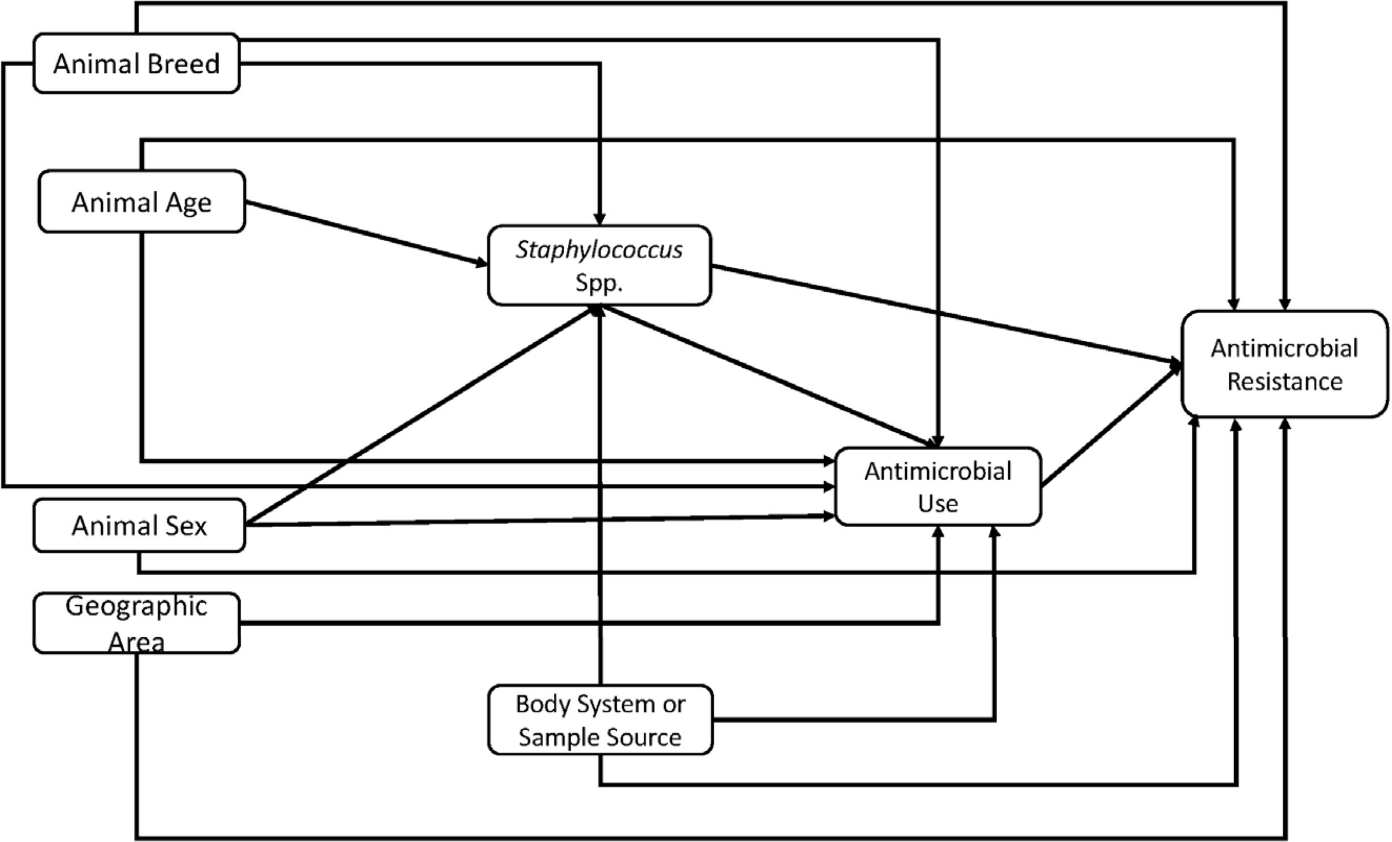
Conceptual model used to guide investigation of predictors of antimicrobial resistance and multidrug resistance among *Staphylococcu*s from canine specimens submitted to the University of Kentucky Veterinary Diagnostic Laboratory, 1993–2009.

The conceptual model used to guide investigation of the predictors of AMR and MDR is shown in Fig 2. Predictors of AMR and MDR were assessed for significant associations with the outcomes of interest (AMR and MDR) in two steps: (1) univariable regression model for each predictor variable listed above were fit to the data and the variable assessed for unadjusted association (using a relaxed α of 0.15) with the outcome variable (either AMR or MDR); and (2) multivariable logistic regression model. Predictor variables with a p≤0.15 in step 1 were included in step 2. The multivariable logistic regression model was built using a manual backwards elimination approach. Only predictor variables that were statistically significant at p≤0.05 were included in the final main effects multivariable logistic regression model. Confounding was assessed by comparing the change in the regression coefficients of the variables in the model with and without the suspected confounder. A variable was considered a confounder and retained in the final model if there was at least a 20% change in the regression coefficients of any of the other variables already in the model. Two-way interaction terms of variables in the final main-effects model were assessed for statistical significance. Odds ratios and their corresponding 95% confidence intervals were calculated for all variables in the final model. Hosmer-Lemeshow goodness of fit test was used to assess the final model.

## Results

A total of 4,972 isolates were included in the final analysis for antimicrobial resistance. Of these 2,667 antimicrobial susceptibility test results (moderately susceptible [n = 2,666] and intermediate [n = 1]) were re-classified as resistant. Of the assessed isolates, 68.1% (3,388/4,972) were SIG, 18.3% (907/4,972) were CoNS, 7.5% (375/4,972) were *S*. *aureus*, 5.8% (290/4,972) were *S*. *hyicus*, and *S*. *schleiferi* subsp. *coagulans* comprised 0.2% (12/4,972) of the isolates (Table 2). Assessment of the distribution of *Staphylococcus* spp. by specimen source revealed that most of the SIG isolates were from skin, hair, and nail specimens (54.1%) followed by ear specimens (24.9%) (Table 2). The most common specimen source was skin, hair and nails (54.1%) followed by ear (24.9%) (Table 2).

**Table 2:** Specimen sources and *Staphylococcus* spp. from canine specimens submitted to the UKVDL^1^, 1993–2009.

Species	Ear	Skin, Hair, & Nails	Urine & Bladder	Mucosal Surfaces	All Others	Total
	% (#)	% (#)	% (#)	% (#)	% (#)	% (#)
*S*. *aureus*	20.5 (77)	48.5 (182)	10.7 (40)	9.3 (35)	10.9 (41)	7.5 (375)
SIG^2^	25.0 (848)	55.8 (1,889)	7.3 (246)	3.9 (131)	8.1 (274)	68.21(3,388)
*S*. *schleiferi* subsp *coagulans*	58.3 (7)	41.7 (5)	-	-	-	0.2 (12)
CoNS^3^	21.8 (198)	53.4 (484)	6.8 (62)	5.5 (50)	12.5 (113)	18.3 (907)
*S*. *hyicus*	37.9 (110)	44.8 (130)	6.6 (19)	2.8 (8)	7.9 (23)	5.8 (290)
**Total**	24.9 (1,240)	54.1 (2,690)	7.4 (367)	4.5 (224)	9.1 (451)	4,972

^1^University of Kentucky Veterinary Diagnostic Laboratory

^2^SIG = *S*. *intermedius* group

^3^CoNS = Coagulase negative *Staphylococcus*

The overall percentage of antimicrobial resistant (AMR) isolates was 77.2% (3,840/4972) (Table 3). There was a significant (p <0.001) unadjusted association between *Staphylococcus* spp. and AMR with CoNS (81.3%; 737/907) having the highest level followed by *S*. *aureus* (80.5%; 302/375) and SIG (77.6%; 2629/3388). *S*. *hyicus* (57.9%; 168/290) and *S*. *schleiferi* subsp. *coagulans* (33.3%; 4/12) had the lowest levels of antimicrobial resistance. The overall level of multidrug resistance (MDR) was 25.6% (Table 3). There was also a significant (p<0.001) unadjusted association between *Staphylococcus* spp. and MDR with the highest levels being observed in *S*. *aureus* (30.1%; 113/375), CoNS (29.4%; 267/907), and SIG 25.4%; (860/3388). The lowest levels of MDR were observed in *S*. *hyicus* (11.7%; 34/290) and *S*. *schleiferi* subsp. *coagulans* (8.3%; 1/12). Overall, 22.8% (1,132/4,972) of the *Staphylococcous* spp. tested showed no resistance to any of the antimicrobials evaluated (Table 3). However, a small percentage showed extensive drug resistance (XDR) with 0.1% (4/4,972) showing resistance to 13 of the 16 antimicrobials tested (Table 3). A small percentage (1.1% [53/4,972]) of *Staphylococcus* isolates (that included all *Staphylococcous* spp. except *S*. *schleiferi* subsp. *coagulans*) showed resistance to 9 antimicrobials (Table 3).

**Table 3:** Antimicrobial resistance of *Staphylococcus* spp. from canine specimens submitted to the UKVDL^1^, 1993–2009.

Number of						
drug isolates	*S*. *aureus*	SIG^2^	*S*. *schleiferi*	CoNS^3^	*S*. *hyicus*	Total
resistant to	% (#)	% (#)	% (#)	% (#)	% (#)	% (#)
0	19.5 (73)	22.4 (759)	66.7 (8)	18.7 (170)	42.1 (122)	22.8 (1,132)
1	26.4 (99)	26.5 (899)	8.3 (1)	26.1 (237)	26.2 (76)	26.4 (1,312)
2	20.0 (75)	20.8 (706)	16.7 (2)	21.6 (196)	17.6 (51)	20.7 (1,030)
3	43 (11.5)	14.6 (495)	8.3 (1)	13.5 (122)	4.8 (14)	13.6 (675)
4	7.5 (28)	6.3 (213)	-	6.8 (62)	2.8 (8)	6.3 (311)
5	3.7 (14)	1.6 (55)	-	3.1 (28)	2.8 (8)	2.1 (105)
6	2.1 (8)	2.2 (74)	-	2.4 (22)	2.1 (6)	2.2 (110)
7	1.1 (4)	2.4 (82)	-	2.3 (21)	1.0 (3)	2.2 (110)
8	2.1 (8)	1.3 (44)	-	3.0 (27)	0.3 (1)	1.6 (80)
9	3.2 (12)	0.9 (31)	-	1.0 (9)	0.3 (1)	1.1 (53)
10	1.1 (4)	0.4 (12)	-	0.7 (6)	-	0.4 (22)
11	1.1 (4)	0.4 (12)	-	0.4 (4)	-	0.4 (20)
12	0.5 (2)	0.1 (4)	-	0.2 (2)	-	0.2 (8)
13	0.3 (1)	0.1 (2)	-	0.1 (1)		0.1 (4)
Total AMR^4^	80.5 (302/375)	77.6 (2,629/3,388)	33.3 (4/12)	81.3 (737/907)	57.9 (168/290)	77.2 (3,840/4,972)
Total MDR^5^	30.1 (113/375)	25.4 (860/3,388)	8.3 (1/12)	29.4 (267/907)	11.7 (34/290)	25.6 (1,275/4,972)

^1^UKVDL = University of Kentucky Veterinary Diagnostic Laboratory

^2^SIG = *S*. *intermedius* Group

^3^CoNS = Coagulase negative *Staphylococcus*

^4^AMR = Antimicrobial Resistance

^5^MDR = Multiple drug resistance

A total of 4,944 isolates were assessed for both oxacillin and lincomycin resistance. Co-resistance to oxacillin was assessed because oxacillin resistance is representative of methicillin resistance and is of clinical importance. Of the isolates assessed for co-resistance to oxacillin and lincomycon, 1.6% (79/4,944) were resistant to both, 6.5% (320/4,944) were susceptible to oxacillin but resistant to lincomycin, and 2.5% (122/4,944) were resistant to oxacillin but susceptible to lincomycin while 89.5% (4,423/4,944) were susceptible to both. Similarly, a total of 4,848 isolates were assessed for both oxacillin and enrofloxacin resistance. Only 1.3% (61/4,848) of these were resistant to both, 0.7% (35/4,848) were susceptible to oxacillin but resistant to enrofloxacin, 2.8% (136/4,848) were resistant to oxacillin but susceptible to enrofloxacin while 95.2% (4,616/4,848) were susceptible to both. When distribution of oxacillin resistance was assessed by species of *Staphylococcus*, resistance to only oxacillin was most common among CoNS (3.8%) followed by *S*. *hyicus* (1.7%) isolates (Table 4). CoNS had the highest (7.9%) proportion of isolates that were both oxacillin resistant and MDR. This was followed by *S*. *aureus* (7.0%) (Table 4). Antimicrobial resistance profiles of all isolates that were resistant to at least one antimicrobial is presented in the supporting/supplementary table (S1 Table).

**Table 4:** Oxacillin-resistant and MDR^1^ *Staphylococcus* spp. from canine specimens submitted to the UKVDL^2^, 1993–2009.

Species	Oxacillin only	Oxacillin and MDR^1^
	% (frequency)	% (frequency)
***S*. *aureus***	0 (0/374)	7.0 (26/374)
**SIG**^**3**^	0.52 (16/3,381)	1.3 (45/3,381)
***S*. *schleiferi* subsp *coagulans***	0 (0/12)	-
**CoNS**^**4**^	3.8 (34/906)	7.9 (72/906)
***S*. *hyicus***	1.7 (5/289)	1.0 (3/289)
**Total**	1.1 (55/4,962)	2.9 (146/4,962)

^1^MDR = Multiple drug resistance

^2^UKVDL = University of Kentucky Veterinary Diagnostic Laboratory

^3^SIG = *S*. *intermedius* group

^4^CoNS = Coagulase negative *Staphylococcus*

### Temporal patterns of resistance

**Fig 3:**
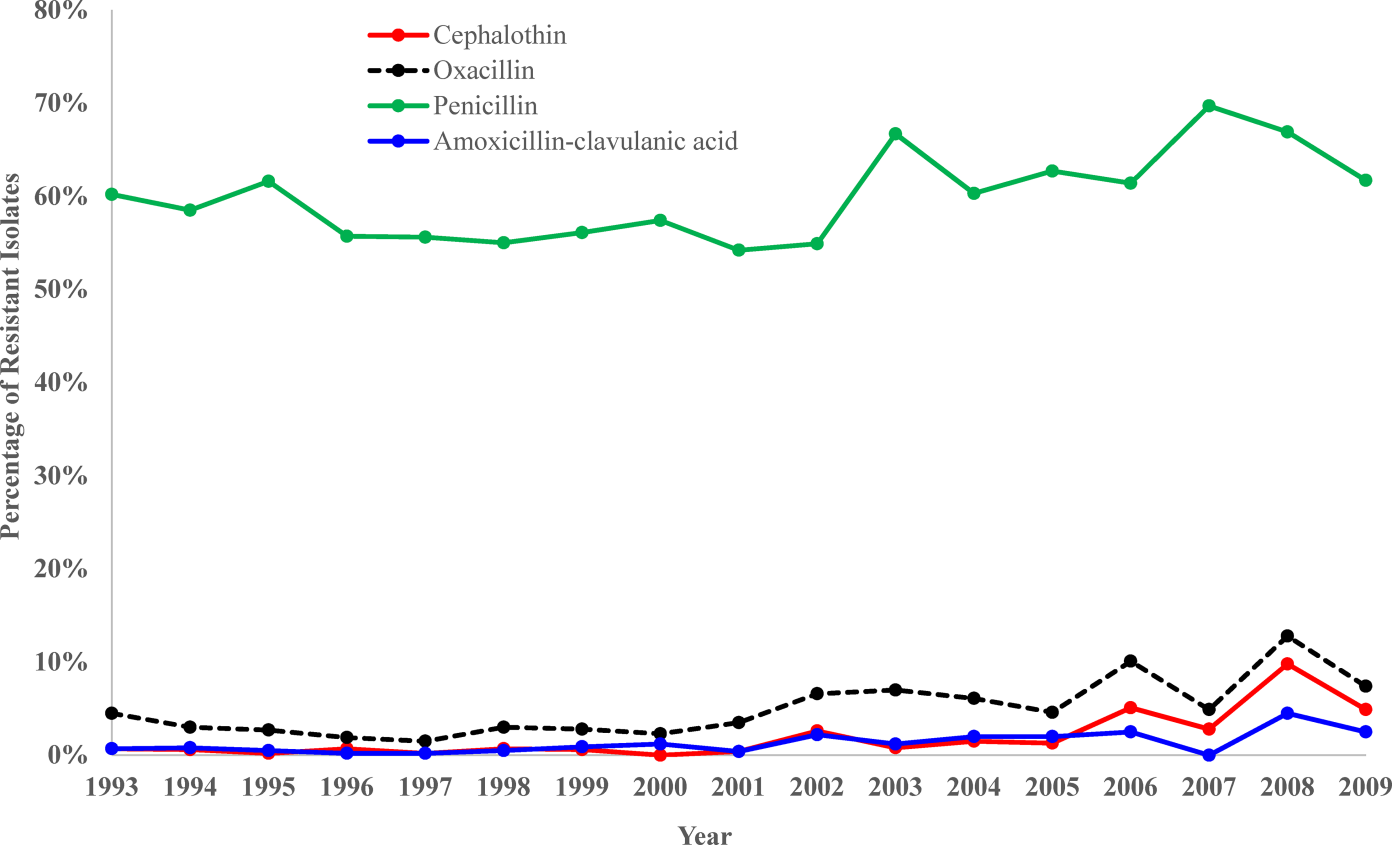
Trends in *Staphylococcus* resistance to β-Lactams among canine specimens submitted to the University of Kentucky Veterinary Diagnostic Laboratory, 1993–2009.

Significant temporal trends were observed among 12 of the 16 antimicrobials covering 6 of the 9 drug classes assessed (Table 5). Isolates showed significant (p<0.05) increasing temporal trends in resistance to amoxicillin-clavulanic acid, cephalothin, oxacillin, and penicillin (Table 5 and Fig 3). Among the β-lactams, isolates exhibited highest levels of resistance to penicillin which ranged from 69.7% (99/142) in 2007 to 54.2% (123/227) in 2001 (Table 5 and Fig 3).

**Table 5:** Trends in AMR^1^ of *Staphylococcus* from canine specimens tested at the UKVDL^2^, 1993–2009.

Group/Antimicrobial	Percentage (number of specimens tested) of resistant isolates to an antimicrobial agent									Total	P-values CAT-T^3^
	1993	1995	1997	1999	2001	2003	2005	2007	2009		
**Aminocoumarin**											
Novobiocin	3.7 (600)	2.2 (402)	2.3 (475)	0.6 (321)	1.8 (226)	1.6 (243)	1.3 (153)	1.4 (142)	1.2 (81)	2.1 (4964)	0.360
**Aminoglycosides**	17.2 (600)	9.7 (402)	8.0 (475)	10.9 (321)	13.7 (227	11.1 (243)	9.8 (153)	11.3 (142)	18.5 (81)	11.7 (4972)	0.514
Gentamicin	2.3 (596)	2.2 (402)	2.1 (475)	3.4 (321)	5.7 (227)	2.5 (243)	5.9 (152)	5.6 (142)	7.4 (81)	3.3 (4965)	**<0.001**
Kanamycin	14.0 (594)	9.5 (402)	7.8 (475)	10.9 (321)	13.7 (227)	10.3 (242)	9.8 (153)	10.6 (142)	18.5 (81)	10.6 (4961)	0.111
Neomycin	5.7 (595)	5.5 (402)	3.8 (475)	3.4 (321)	7.0 (227)	7.4 (242)	6.5 (153)	7.7 (142)	12.3 (81)	5.9 (4965)	**<0.001**
Streptomycin	15.2 (597)	7.9 (114)	0	*	*	*	*	*	*	14.9 (1231)	**<0.001**
**Beta-Lactams**	61.3 (600)	61.8 (402)	56.0 (475)	56.4 (321)	55.1 (227)	68.3 (243)	63.4 (153)	69.7 (142)	61.7 (81)	59.7 (4972)	**0.010**
Amoxicillin-clavulanic acid	0.7 (594)	0.5 (402)	0.2 (475)	0.9 (321)	0.4 (224)	1.2 (242)	2.0 (152)	0.0 (140)	2.5 (81)	1.0 (4952)	**<0.001**
Cephalothin^4^	0.7 (596)	0.2 (402)	0.2 (475)	0.6 (321)	0.4 (227)	0.8 (243)	1.3 (153)	2.8 (142)	4.9 (81)	1.2 (4968)	**<0.001**
Oxacillin	4.5 (599)	2.7 (402)	1.5 (475)	2.8 (321)	3.5 (227)	7.0 (242)	4.6 (153)	4.9 (142)	7.4 (81)	4.1 (4962)	**<0.001**
Penicillin	60.2 (598)	61.6 (401)	55.6 (475)	56.1 (321)	54.2 (227)	66.7 (243)	62.7 (153)	69.7 (142)	61.7 (81)	58.8 (4966)	**0.024**
**Fluoroquinolones**											
Enrofloxacin	0.7 (538)	0.2 (402)	0.4 (474)	1.6 (320)	1.8 (226)	3.7 (241)	3.3 (152)	6.4 (140)	7.4 (81)	2.0 (4856)	**<0.001**
**Lincosamides**											
Lincomycin	11.1 (594)	7.2 (402)	6.3 (475)	6.5 (321)	7.2 (223)	10.3 (242)	6.5 (153)	7.7 (142)	16.0 (81)	8.1 (4949)	0.490
**Macrolides**											
Erythromycin	11.8 (600)	7.5 (402)	7.4 (475)	8.1 (321)	9.3 (227)	12.8 (243)	10.5 (152)	8.5 (142)	16.0 (81)	9.5 (4961)	0.124
**Polypeptides**											
Bacitracin	9.1 (596)	3.0 (402)	0.8 (474)	2.2 (321)	4.0 (227)	3.7 (243)	2.6 (153)	0.0 (140)	2.5 (81)	3.9 (4960)	**0.002**
**Sulfonamides**	74.4 (597)	61.7 (402)	51.8 (475)	45.5 (321)	46.3 (227)	44.0 (243)	44.4 (153)	43.0 (142)	37.0 (81)	54.0 (4969)	**<0.001**
Sulfadiazine-Trimethoprim	74.4 (597)	61.4 (402)	51.5 (474)	44.5 (319)	44.9 (227)	43.6 (243)	43.1 (153)	43.0 (142)	35.8 (81)	53.4 (4962)	**<0.001**
Sulfamethoxazole	18.5 (596)	16.4 (402)	6.9 (475)	10.3 (321)	15.9 (227)	22.6 (243)	15.7 (153)	12.0 (142)	13.6 (81)	15.1 (4965)	0.570
**Tetracycline**											
Tetracycline	31.8 (598)	26.9 (401)	24.0 (475)	23.9 (318)	24.6 (224)	23.9 (238)	19.1 (152)	28.1 (139)	20.3 (79)	25.1 (4941)	**0.010**
**AMR**^**1**^	88.7 (600)	79.1 (402)	73.5 (475)	72 (321)	71.4 (227	77.4 (243)	73.9 (153)	78.2 (142)	70.4 (81)	77.2 (4972)	**<0.001**
**MDR**^**5**^	34.3 (600)	28.1 (402)	22.1 (475)	20.6 (321)	26.0 (227)	24.3 (243)	22.2 (153)	21.8 (142)	27.2 (81)	25.6 (4972)	**<0.001**

^1^AMR: Antimicrobial Resistance

^2^UKVDL: University of Kentucky Veterinary Diagnostic Laboratory

^3^P-Values of CAT-T: Cochran-Armitage trend test

^4^Cephalothin: Cephalosporin I

^5^Multidrug Resistance

*Numbers suppressed because of very small specimen sample sizes (unreliable estimates)

**Fig 4:**
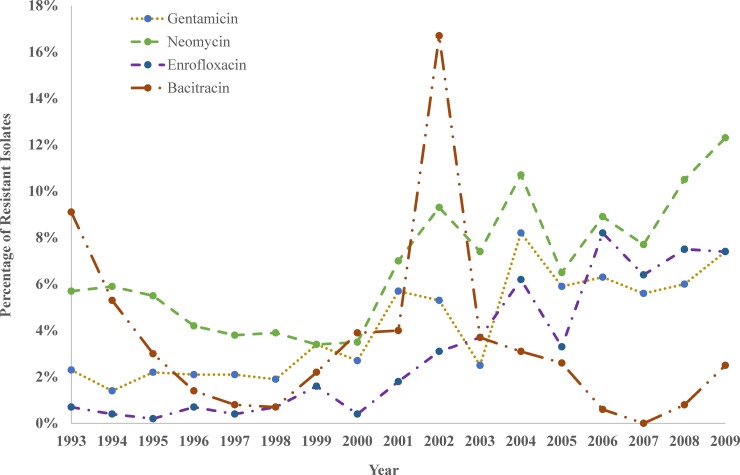
Trends in *Staphylococcus* resistance to aminoglycosides, fluoroquinolones and polypeptide among canine specimens submitted to the University of Kentucky Veterinary Diagnostic Laboratory, 1993–2009.

There was significant (p<0.001) increasing temporal trend in resistance to 3 of the 4 aminoglycosides tested (Table 5 and Fig 4). Overall 14.9% of the 1,231 specimens tested for streptomycin susceptibility were resistant. However, the annual changes in resistance to streptomycin from 1997 to 2009 were based on very small numbers (< 5) and hence have been suppressed on Table 5. Overall, 3.3% and 5.9% of the 4,965 isolates tested showed resistance to gentamicin and neomycin, respectively.

Only one antimicrobial in each of the fluoroquinolone and polypeptide classes were tested. Both enrofloxacin (p<0.001) and bacitracin (p = 0.002) showed significant temporal trends (Table 5 and Fig 4). Enrofloxacin resistant isolates showed a significant increasing temporal trend. Overall resistance was 2.0% (96/4856) but ranged from 0.2% (1/402) in 1995 to 8.2% (13/158) in 2006. Bacitracin resistant isolates showed a decreasing temporal trend until 1999 (2.2% [7/321]) with a major spike in 2002 (16.7% [38/227]) and then decreased again in 2003 (3.7% [9/243]).

**Fig 5:**
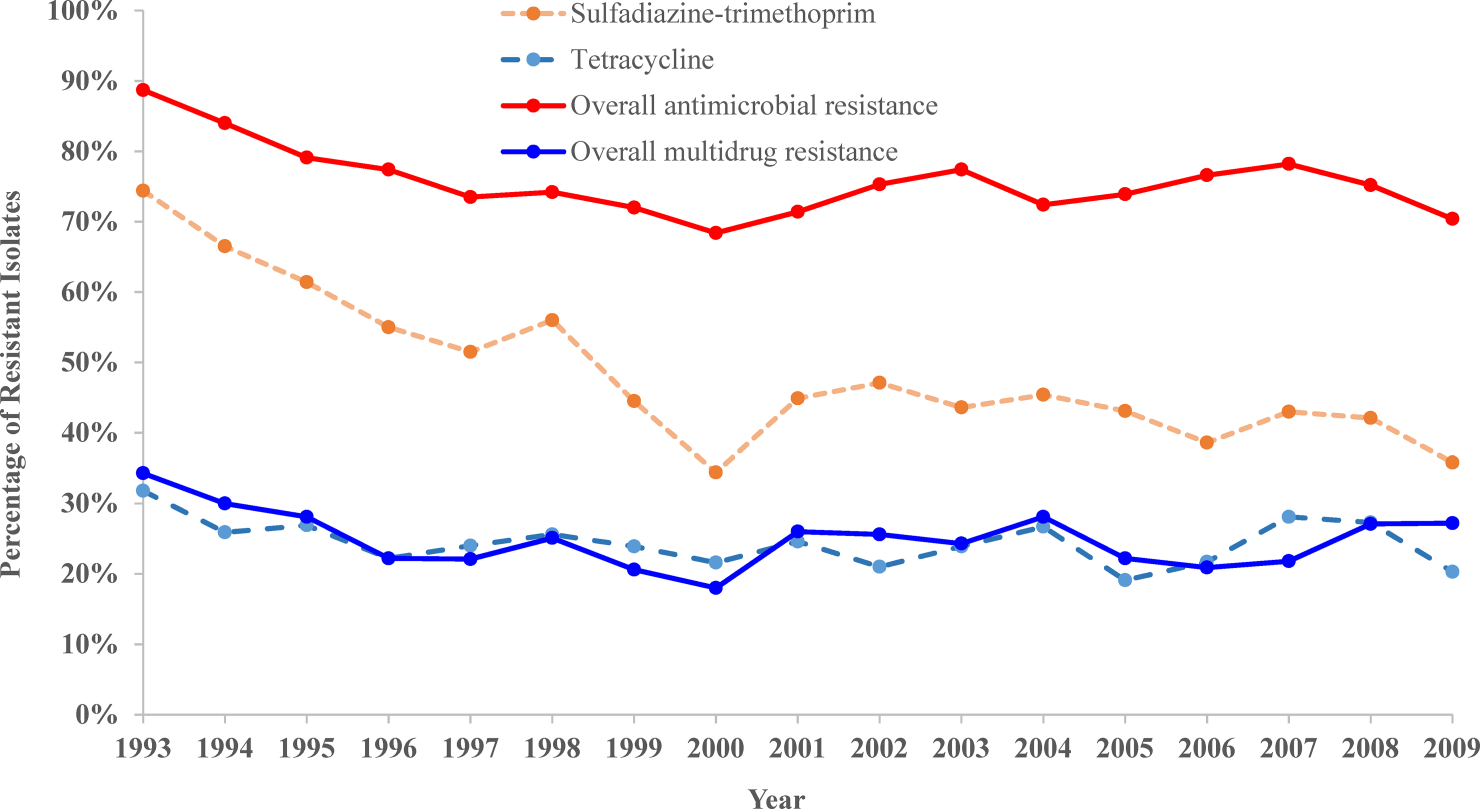
Trends in *Staphylococcus* resistance to sulfonamides and tetracycline as well as overall antimicrobial and multi-drug resistance among canine specimens submitted to the University of Kentucky Veterinary Diagnostic Laboratory, 1993–2009.

The sulfadiazin (p<0.001) and tetracycline (p = 0.010) antimicrobial classes both showed moderately high levels of resistance with a significant overall decreasing temporal trend (Table 5 and Fig 5). Overall *Staphylococcus* resistance to sulfadiazin was 53.4% (2,648/4,962) but ranged from 34.4% (88/256) in 2000 to 74.4% (444/597) in 1993. The overall level of resistance to tetracycline was 25.1% (1,241/4,941) and ranged from 19.1% (29/152) in 2005 to 31.8% (190/598) in 1993. Finally, there was a significant decreasing temporal trend in overall AMR and MDR (Table 5 and Fig 5).

### Predictors of AMR and MDR

Sample distribution across the predictor variables assessed were: *Staphlylococcus* spp. (n = 4,972), geographic region (n = 4,972), AKC breed categories (n = 4,275), age groups (3,857), sex (n = 4,780), and specimen source (n = 4,972). A total of 697 records had missing breed information while 192 and 1,115 records had missing sex and age group information, respectively. One case was also eliminated from age category due to an implausible age designation (85 years). Based on an α = 0.15, there were significant unadjusted associations between AMR and 5 of the 6 potential predictor variables investigated in the unadjusted logistic models: *Staphylococcus* spp. (p<0.001), geographic region (p<0.001), AKC breed categories (p = 0.015), age group (p = 0.002), and specimen source (p<0.001) (Table 6). Based on the final multiple logistic regression model AMR had significant association with *Staphylococcus* spp. (p<0.001), geographic region (p = 0.001), and specimen source (p<0.001) (Table 7). The odds of AMR were significantly higher among *S*. *aureus* (OR: 2.728, 95% CI: 1.923–3.872), SIG (OR: 2.422, 95% CI: 1.887–3.109), and CoNS (OR: 3.009, 95% CI: 2.251–4.022) compared to *S*. *hyicus*. With respect to the geographical effects, the odds of AMR was significantly higher in Region 3 (OR: 2.041, 95% CI: 1.398–2.978) and Region 5 (OR: 1.813, 95% CI: 1.303–2.523) than Region 4. For specimen source, the odds of AMR was significantly higher among specimens from skin, hair, and nails (OR: 1.330, 95% CI: 1.138–1.555) as well as urine and bladder (OR: 1.870, 95% CI: 1.371–2.549), mucosal surfaces (OR: 2.613, 95% CI: 1.705–4.004), and all others (OR: 1.372, 95% CI: 1.058–1.779) compared to ear specimens.

**Table 6:** Unadjusted associations of *Staphylococcus* AMR^1^ and predictors among specimens submitted to UKVDL^2^, 1993–2009.

		AMR^1^		Unadjusted			
Predictor	Total No	#	%	OR^3^	95% CI^4^		P-value
***Staphylococcus***							**<0.001**
*S*. *aureus*	375	302	80.5	3.004	2.126	4.246	**<0.001**
*S*. *intermedius* group	3388	2629	77.6	2.515	1.965	3.219	**<0.001**
*S*. *schleiferi* subsp *coagulans*	12	4	33.3	0.363	0.107	1.233	0.104
CoNS^5^	907	737	81.3	3.148	2.364	4.193	**<0.001**
*S*. *hyicus*	290	168	57.9	-	-	-	-
**Geographic Region**^**6**^							**<0.001**
Region 2	2	1	50.0	0.531	0.033	8.640	0.656
Region 3	672	548	81.5	2.347	1.623	3.393	**<0.001**
Region 5	3597	2802	77.9	1.871	1.355	2.585	**<0.001**
Region 6	238	167	70.2	1.249	0.822	1.898	0.298
Region 7	98	69	70.4	1.263	0.740	2.157	0.392
Region 8	192	140	72.9	1.430	0.915	2.234	0.117
Region 4	173	113	65.3	-	-	-	-
**AKC Breed categories**							**0.015**
Herding	338	253	74.9	1.031	0.779	1.366	0.829
Hound	482	384	79.7	1.358	1.047	1.761	**0.021**
Mixed breed	900	681	75.7	1.078	0.879	1.322	0.473
Non-Sporting	555	440	79.3	1.326	1.037	1.696	**0.025**
Terrier	339	268	79.1	1.308	0.974	1.757	0.074
Toy	240	181	75.4	1.063	0.769	1.470	0.711
Working	333	276	82.9	1.678	1.223	2.301	**0.001**
Sporting	1088	808	74.3	-	-	-	-
**Age Groups**							**0.002**
<2 years	683	557	81.6	1.339	1.052	1.704	**0.018**
2–4 years	979	774	79.1	1.144	0.926	1.412	0.212
4–6 years	611	453	74.1	0.868	0.689	1.094	0.232
6–8 years	543	398	73.3	0.831	0.655	1.055	0.129
>8 years	1041	799	76.8	-	-	-	-
**Sex**							0.238
Female	2569	2007	78.1	1.087	0.949	1.245	0.228
Male	2210	1695	76.7	-	-	-	-
**Specimen Source**							**<0.001**
Skin, Hair, Nails	2,690	2098	78.0	1.422	1.220	1.657	**<0.001**
Urine, Bladder	367	308	83.9	2.094	1.544	2.840	**<0.001**
Mucosal Surfaces	224	197	87.4	2.927	1.922	4.457	**<0.001**
All Others	451	352	78.0	1.426	1.106	1.840	**0.006**
Ear	1,240	885	71.4	-	-	-	-

^1^AMR = Antimicrobial resistance

^2^UKVDL = University of Kentucky Veterinary Diagnostic Laboratory

^3^OR = Odds Ratio

^4^95% CI = 95% Confidence Interval

^5^CoNS = Coagulase negative *Staphylococcus*

^6^No specimens were submitted from Region 1

**Table 7:** Final model showing adjusted associations of *Staphylococcus* AMR^1^ and its predictors among specimens submitted to UKVDL^2^, 1993–2009.

Predictor	Total No.	Adjusted OR^3^	95% CI^4^		P-value
***Staphylococcus***					**<0.001**
*S*. *aureus*	375	2.728	1.923	3.872	**<0.001**
*S*. *intermedius* group	3,388	2.422	1.887	3.109	**<0.001**
*S*. *schleiferi* subsp *coagulans*	12	0.386	0.113	1.319	0.129
CoNS^5^	907	3.009	2.251	4.022	**<0.001**
*S*. *hyicus*	290	-	-	-	-
**Geographic Region**^**6**^					**<0.001**
Region 2	2	0.474	0.029	7.738	0.600
Region 3	672	2.041	1.398	2.978	**<0.001**
Region 5	3,597	1.813	1.303	2.523	**<0.001**
Region 6	238	1.222	0.797	1.874	0.357
Region 7	98	1.108	0.645	1.904	0.711
Region 8	192	1.357	0.861	2.138	0.188
Region 4	173	-	-	-	-
**Specimen Source**					**<0.001**
Skin, Hair, Nails	2,690	1.330	1.138	1.555	**<0.001**
UrineBladder	367	1.870	1.371	2.549	**<0.001**
Mucosal Surfaces	224	2.613	1.705	4.004	**<0.001**
All Others	451	1.372	1.058	1.779	**0.017**
Ears	1,240	-	-	-	-

^1^AMR = Antimicrobial resistance

^2^UKVDL = University of Kentucky Veterinary Diagnostic Laboratory

^3^OR = Odds Ratio

^4^95% CI = 95% Confidence Interval

^5^CoNS = Coagulase negative *Staphylococcus*

^6^No specimens were submitted from Region 1

**Fig 6:**
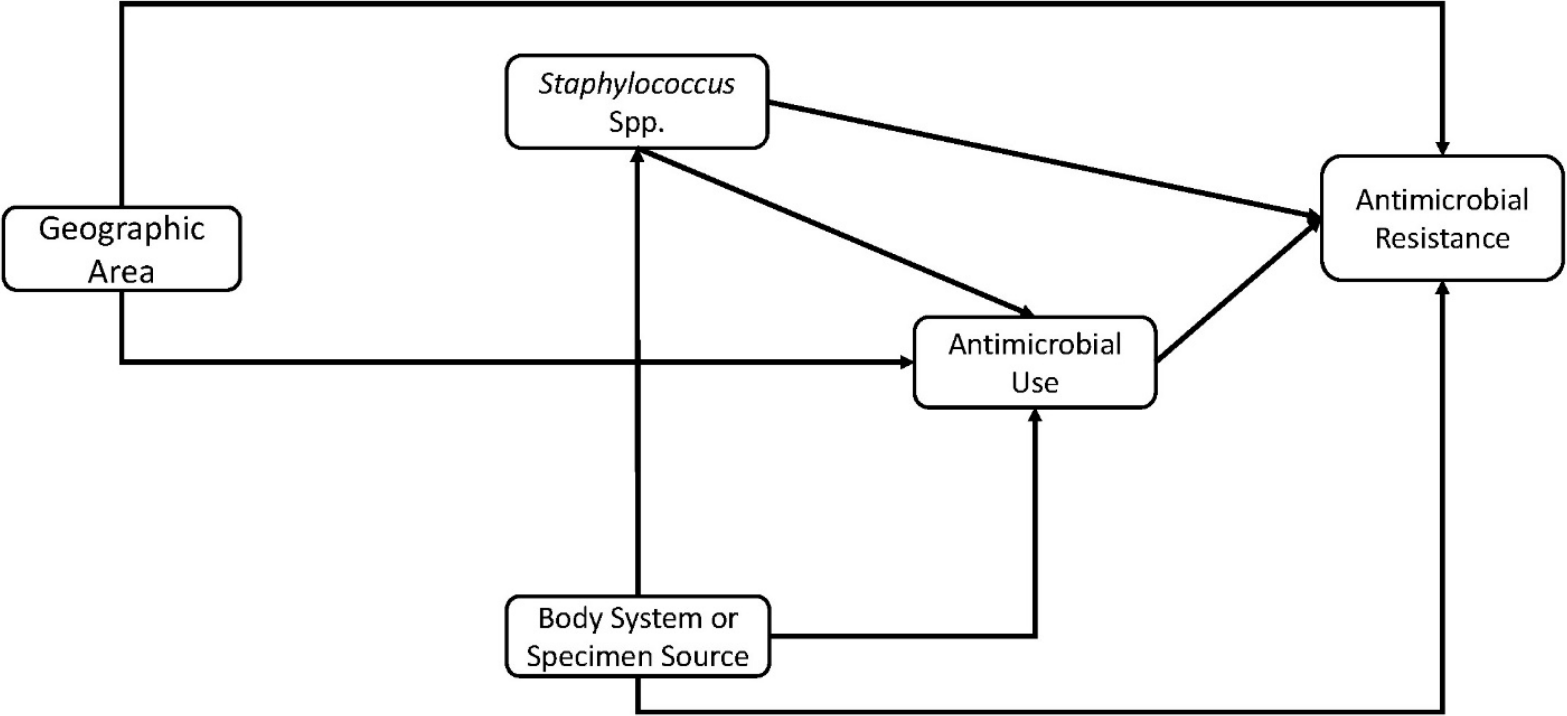
Conceptual model based on significant predictors of antimicrobial resistance and multidrug resistance among *Staphylococcu*s from canine samples submitted to the University of Kentucky Veterinary Diagnostic Laboratory, 1993–2009.

For MDR, 5 of the 6 potential predictor variables investigated for unadjusted associations using a relaxed α = 0.15 were significant: *Staphylococcus* species (p<0.001), geographic region (p = 0.001), AKC breed categories (p = 0.014), age group (p = 0.111), and specimen source (p<0.001) (Table 8). Based on the final multiple logistic regression model, MDR had significant associations with *Staphylococcus* spp. (p<0.001), geographic region (p = 0.007), and specimen source (p<0.001) (Table 9). The odds of MDR was significantly higher among *S*. *aureus* (OR: 3.001, 95% CI: 1.966–4.580), SIG (OR: 2.452, 95% CI: 1.698–3.541), and CoNS (OR: 2.993, 95% CI: 2.032–4.4093) isolates than *S*. *hyicus* isolates. Region 3 (OR: 2.003, 95% CI: 1.272–3.156), Region 5 (OR: 1.886, 95% CI: 1.230–2.891), and Region 7 (OR: 2.480, 95% CI: 1.365–4.505) had significantly higher odds of MDR compared to Region 4. Similarly, isolates obtained from skin, hair, and nails (OR: 1.265, 95% CI: 1.075–1.489) as well as urine and bladder (OR: 1.790, 95% CI: 1.381–2.321) and mucosal surface (OR: 1.651, 95% CI: 1.202–2.268) isolates had significantly higher odds of MDR compared to isolates from the ear specimens. The revised conceptual model based on the significant variables in the models used to identify predictors of AMR and MDR is shown in Fig 6.

**Table 8:** Unadjusted associations of *Staphylococcus* MDR^1^ and predictors among specimens submitted to UKVDL^2^, 1993–2009.

Predictor		MDR^1^					
	Total No.	#	%	OR^3^	95% CI^4^		P-value
***Staphylococcus***							**<0.001**
*S*. *aureus*	375	113	30.1	3.247	2.133	4.944	**<0.001**
*S*. *intermedius* group	3388	860	25.4	2.561	1.776	3.694	**<0.001**
*S*. *schleiferi* subsp *coagulans*	12	1	8.3	0.684	0.086	5.469	0.721
CoNS^5^	907	267	29.4	3.141	2.137	4.617	**<0.001**
*S*. *hyicus*	290	34	11.7	-	-	-	-
**Geographic Region**^**6**^							**0.001**
Region 2	2	0	0	1	N/A	N/A	N/A
Region 3	672	194	28.9	2.295	1.464	3.596	**<0.001**
Region 5	3597	935	26.0	1.986	1.300	3.033	**0.002**
Region 6	238	44	18.5	1.282	0.755	2.179	0.358
Region 7	98	32	32.7	2.741	1.514	4.962	**0.001**
Region 8	192	44	22.9	1.681	0.984	2.872	0.058
Region 4	173	26	15.0	-	-	-	-
**AKC Breed categories**							**0.014**
Herding	338	90	26.6	1.282	0.969	1.698	0.082
Hound	482	121	25.1	1.184	0.922	1.522	0.186
Mixed breed	900	205	22.8	1.042	0.843	1.288	0.702
Non-Sporting	555	162	29.2	1.456	1.154	1.838	**0.002**
Terrier	339	91	26.8	1.297	0.980	1.715	0.069
Toy	240	69	28.8	1.426	1.041	1.952	**0.027**
Working	333	95	28.5	1.410	1.068	1.862	**0.015**
Sporting	1088	240	22.1	-	-	-	-
**Age Groups**							**0.111**
<2 years	683	181	26.5	1.004	0.807	1.250	0.969
2–4 years	979	244	24.9	0.925	0.757	1.129	0.443
4–6 years	611	136	22.3	0.798	0.630	1.009	0.059
6–8 years	543	157	28.9	1.133	0.899	1.427	0.290
>8 years	1041	275	26.4	-	-	-	-
**Sex**							0.207
Female	2569	680	26.5	1.090	0.957	1.241	0.196
Male	2211	549	24.8	-	-	-	-
**Specimen Source**							**<0.001**
Skin, Hair, Nails	2,690	702	26.1	1.325	1.127	1.557	**0.001**
Urine and Bladder	367	126	34.3	1.961	1.520	2.531	**<0.001**
Mucosal Surfaces	224	73	32.6	1.813	1.329	2.475	**<0.001**
All Others	451	113	25.1	1.254	0.974	1.615	0.079
Ears	1,240	261	21.0	-	-	-	-

^1^MDR = Multiple drug resistance

^2^UKVDL = University of Kentucky Veterinary Diagnostic Laboratory

^3^OR = Odds Ratio

^4^95% CI = 95% Confidence Interval

^5^CoNS = Coagulase negative *Staphylococcus*

^6^No specimens were submitted from Region 1

**Table 9:** Final model of predictors of MDR^1^ among *Staphylococcus* isolates from specimens submitted to the UKVDL^2^, 1993–2009.

Predictor	Total No.	OR^3^	95% CI^4^		P-value
***Staphylococcus***					**<0.001**
*S*. *aureus*	375	3.001	1.966	4.580	**<0.001**
*S*. *intermedius* group	3388	2.452	1.698	3.541	**<0.001**
*S*. *schleiferi* subsp *coagulans*	12	0.727	0.091	5.823	0.764
CoNS^5^	907	2.993	2.032	4.409	**<0.001**
*S*. *hyicus*	290	-	-	-	-
**Geographic Region**^**6**^					**0.007**
Region 2	2	1	N/A	N/A	N/A
Region 3	672	2.003	1.272	3.156	**0.003**
Region 5	3597	1.886	1.230	2.891	**0.004**
Region 6	238	1.237	0.725	2.112	0.436
Region 7	98	2.480	1.365	4.505	**0.003**
Region 8	192	1.583	0.923	2.716	0.095
Region 4	173	-	-	-	-
**Specimen Source**					**<0.001**
Skin, Hair, Nails	2,690	1.265	1.075	1.489	**0.005**
Urine, Bladder	367	1.790	1.381	2.321	**<0.001**
Mucosal Surfaces	224	1.651	1.202	2.268	**0.002**
All Others	451	1.187	0.919	1.534	0.189
Ears	1,240	-	-	-	-

^1^MDR = Multiple drug resistance

^2^UKVDL = University of Kentucky Veterinary Diagnostic Laboratory

^3^OR = Odds Ratio

^4^95% CI = 95% Confidence Interval

^5^CoNS = Coagulase negative *Staphylococcus*

^6^No specimens were submitted from Region 1

## Discussion

This study investigated temporal patterns and predictors of *Staphylococcus* spp. from canine clinical specimens submitted to the University of Kentucky Veterinary Diagnostic Laboratory. The level of AMR observed in this study (77.2%) was lower than the 88% reported by Hauschild and Wójcik [8] in Poland, 90.9% reported by Lilenbaum *et al* [21] in Canada, or the 90.5% reported by Qekwana *et al* [22] in South Africa. However, consistent with findings from previous studies [6,44], SIG was the most common isolate identified and CoNS were the second most common. Similar to previous studies [22,26,27], *S*. *aureus* had the highest levels of AMR and MDR followed by the CoNS [1,45,46]. Unfortunately, in this study, CoNS were not identified to species level nor was testing for *mecA* gene done since it was not part of the diagnostic protocol used by the laboratory that provided the study data. Characterization of CoNS could aid in understanding their clinical relevance, help prevent hospital acquired infections, guide optimal antimicrobial therapy, and aid in understanding transfer of resistance factors from CoNS to CoPS [24,47,48]. The implication of not testing for *mecA* gene is potential under-estimation of levels of resistance to all β-lactams since presence of *mecA* implies resistance to all β-lactams and not just oxacillin.

In this study, 80 of the *Staphylococcus* spp. isolated showed resistance to half (8 out of 16) of the antimicrobials tested while 8 isolates showed resistance to 75% (12 out of 16) of the antimicrobials tested. Although these numbers are relatively small compared to the number of isolates investigated in the study, these findings raise both public health and veterinary medical concerns due to the zoonotic potential and possible transfer of resistance genes among *Staphylococcus* spp. [46]. Moreover, it may be indicative of possible development of XDR over time that could make treatment options more challenging [42,49].

The AMR trends observed in this study illustrate the importance of evaluating individual antimicrobial temporal patterns within large drug classes such as the β-lactams and aminoglycosides. For instance, although the *Staphylococcus* spp. isolates did not show evidence of significant temporal trends in AMR to aminoglycosides (p = 0.514), several individual antimicrobials tested within this class showed significant increasing temporal trend in AMR. Thus, if the analysis had only been performed at the antimicrobial class level, important AMR temporal trends would have been missed. The importance of evaluating individual antimicrobials is also highlighted by the observed varying temporal trends of overall AMR and MDR. The varying trends observed among individual drugs, with some showing increasing while others showed decreasing temporal trends, resulted in overall significant decreasing temporal trends in both AMR and MDR. Additionally, evaluation of the individual drugs also revealed that overall AMR of *Staphylococcus* spp. isolates to β-lactams were relatively low among oxacillin (4.1%), amoxicillin-clavulanic acid (1.0%), and cephalothin (1.2%) while natural penicillin had a consistently higher level of resistance (58.8%) resulting in the overall relatively high AMR of β-lactams. Enrofloxacin (2.0%) and lincosamide (8.1%) also had relatively low levels of resistance among *Staphylococcus* spp. isolates. This has important clinical implications because β-lactams, such as cephalexin and cefpodoxime, as well as enrofloxacin and lincosamide are routinely used in the management of canine allergic dermatitis and pyoderma [50,51]. However, it is worth noting that other studies have reported higher levels of resistance to β-lactams most likely due to selection pressure resulting from higher frequency of drug usage in the concerned populations [10,26,27,52].

It is interesting that although sulfonamides (54.0%) and tetracyclines (25.1%) showed relatively higher levels of AMR than the other drugs, they exhibited significant decreasing temporal trends in AMR over the study period. The observed decline in AMR to both drugs may be due to a decline in usage frequency because of decreasing clinical efficacy. This could result in lower selection pressure and the observed decreasing temporal trend in AMR. Suffice it to say that this finding suggests that tracking individual drug usage preferences over time among clinical veterinarians may be important. Prescott *et al* [53] evaluated antimicrobial resistance to CoPS isolated from canine urinary tract infections and found both increasing and decreasing temporal trends that coincided with shifts in antimicrobial usage within the Veterinary Teaching Hospital. In light of this, we suggest that the following factors be considered when evaluating patterns/changes in AMR: amount of specimens submitted, drug preferences for treatment of specific body systems/conditions, and shifts in available drugs and usage patterns.

Significant predictors of AMR were *Staphylococcus* species, specimen source, and geographical region. Geographic region, as a predictor of AMR, has not been thoroughly investigated in previous studies. In this study we used geographic regions, adopted from CMS rating areas, because they are based on: (a) population homogeneity such as socioeconomic status and population density, and (b) established regions that would be important for repeatability in future studies. Our study findings suggest that higher levels of AMR occur in more urban areas. Region 3 (Louisville, KY) and 5 (Lexington, KY) had the highest rates of submissions as well as high levels of AMR which are not surprising since they have the 2 largest cities with the highest populations in Kentucky [40]. Moreover, Jefferson (Louisville, KY) and Fayette (Lexington, KY) counties have been found to have a higher population of dogs [54]. Thus, we hypothesize that antimicrobial usage rates might be higher in these regions resulting in higher selection pressure and hence higher levels of AMR. This could imply that those living in urban areas may be more likely to approve specimen submissions to diagnostic laboratories. Additionally, high specimen submissions in urban areas may also be due to higher client income, perceived benefits of culture and antimicrobial susceptibility testing, and dynamics in the client-veterinarian relationship leading to tests being offered more frequently.

Regions 7, 8, and portions of 4 are rural Appalachian counties [55]. It has been shown in previous public health studies that populations living in Appalachian counties perceive the value of health care differently leading to increased health disparities among people [56]. This may be the case among pet populations as well. Furthermore, the rural regions tend to have smaller populations, fewer dogs, and hence fewer specimen submissions possibly due to financial limitations and distance to diagnostic laboratories that are usually located in urban areas. Additionally, fewer dogs in rural areas may imply less antimicrobial usage, less selection pressure and hence lower AMR levels. However, more detailed investigations are obviously warranted to identify specific factors responsible for the observed geographic patterns in AMR.

The significant association observed between AMR and *Staphylococcus* species as well as specimen source is consistent with findings from a study by Hoekstra and Paulton [10]. Although the study by Hoekstra and Paulton also found this association between AMR and sex as well as age of the animal, our study did not find these associations. It is worth noting that a South African study by Qekwana *et al* [22] investigated similar predictors of AMR/MDR and did not find a significant association between any of the factors investigated. This may imply that: (a) the importance of these predictors may be dependent on geographical location and population of animals under investigation; (b) testing for AMR vary by geography. Therefore, these issues should always be borne in mind when making comparisons between studies.

This being a retrospective study has some inherent limitations. For instance, the oxacillin-resistant isolates were not checked for *mecA* as this was not part of the diagnostic procedure of the laboratory that supplied the study data. Additionally, no antimicrobial use history was available and therefore we could not assess its association with AMR. Moreover, submission rates to the diagnostic laboratory dramatically decreased over the 16 year study period resulting in a smaller number of yearly isolates tested for antimicrobial resistance. Decreased specimen submissions may have been due to laboratory pricing changes. Additionally, zone diameters for each isolate were not recorded making retrospective changes in break-points to assess their impact on results impossible. In 2009, the *S*. *pseudintermedius* oxacillin zone diameter for resistance changed from 10 to 17. Although this happened during the last year of our study period, it might have led to underestimation of oxacillin resistance in our study. During the study period, the lab used disk diffusion test that would make it more difficult to identify smaller changes in trends compared MIC method. Clinical submissions to diagnostic labs tend to be triggered by a failure of response to empirical therapy and would potentially result in overestimation of resistance levels in the population. Finally, issues of sample size precluded some secondary sub-analyses.

## Conclusion

The above limitations notwithstanding, the study provides some useful epidemiological information to guide future studies. It is evident that temporal patterns in *Staphylococcus* spp. resistance varied greatly across antimicrobials. This highlights the need for such investigations to be carried out for specific drugs as opposed to performing the analysis for entire drug classes, or worse still, all the drugs combined. The significant association between both AMR and MDR with geographic region may suggest that local factors play a role in the problem and will require further investigations.

## Supporting information

S1 Table Resistance profiles of canine Staphylococcus specimens submitted to UKVDL1 from 1993–2009. (DOCX)
