# Supplementary material for: Temporal trends and predictors of antimicrobial resistance among Staphylococcus spp. isolated from canine specimens submitted to a diagnostic laboratory
Source: PLoS One. 2018 Aug 1;13(8):e0200719. doi: 10.1371/journal.pone.0200719 (PMC6070192; doi:10.1371/journal.pone.0200719)
Supplement: S1 Table — (DOCX) [file pone.0200719.s001.docx]

**S1 Table: Resistance profiles of canine *Staphylococcus* specimens submitted to UKVDL^1^ from 1993-2009.**

| **Abx #** | **Resistance Profile** | **freq** | **%** |
| --- | --- | --- | --- |
| 13 | aug^2^-bac^3^-cep^4^-enr^5^-ery^6^-gen^7^-kan^8^-lin^9^-neo^10^-oxa^11^- pen^12^-tet^16^-tri^17^ | 2 | 0.002 |
| 13 | aug-cep-enr-ery-gen-kan-lin-neo-oxa-pen-str^14^-sul^15^-tri | 2 | 0.002 |
| 12 | aug-bac-cep-enr-ery-gen-kan-oxa-pen-sul-tet-tri | 1 | 0.001 |
| 12 | aug-bac-cep-ery-gen-kan-lin-oxa-pen-str-sul-tri | 1 | 0.001 |
| 12 | aug-bac-enr-ery-gen-kan-lin-neo-oxa-pen-sul-tri | 1 | 0.001 |
| 12 | aug-enr-ery-gen-kan-lin-neo-oxa-pen-sul-tet-tri | 1 | 0.001 |
| 12 | cep-enr-ery-gen-kan-lin-neo-oxa-pen-str-sul-tri | 1 | 0.001 |
| 12 | cep-enr-ery-gen-kan-lin-neo-oxa-pen-sul-tet-tri | 3 | 0.003 |
| 11 | aug-bac-cep-enr-ery-gen-kan-lin-oxa-pen-tet | 1 | 0.001 |
| 11 | aug-bac-cep-enr-kan-lin-oxa-pen-str-sul-tri | 1 | 0.001 |
| 11 | aug-cep-enr-ery-gen-kan-lin-oxa-pen-sul-tri | 1 | 0.001 |
| 11 | aug-cep-enr-ery-gen-kan-neo-oxa-pen-tet-tri | 1 | 0.001 |
| 11 | aug-cep-ery-gen-kan-lin-neo-pen-str-sul-tet | 1 | 0.001 |
| 11 | aug-cep-ery-gen-kan-lin-oxa-pen-sul-tet-tri | 1 | 0.001 |
| 11 | aug-cep-ery-kan-lin-neo-oxa-pen-str-sul-tet | 1 | 0.001 |
| 11 | aug-enr-ery-gen-kan-lin-neo-oxa-pen-sul-tri | 1 | 0.001 |
| 11 | cep-enr-ery-gen-kan-neo-nov-oxa-pen-sul-tet | 1 | 0.001 |
| 11 | cep-enr-ery-kan-lin-neo-oxa-pen-sul-tet-tri | 1 | 0.001 |
| 11 | cep-ery-gen-kan-lin-neo-nov-pen-str-sul-tet | 2 | 0.002 |
| 11 | cep-ery-gen-kan-lin-neo-oxa-pen-sul-tet-tri | 1 | 0.001 |
| 11 | enr-ery-gen-kan-lin-neo-oxa-pen-str-sul-tri | 1 | 0.001 |
| 11 | enr-ery-gen-kan-lin-neo-oxa-pen-sul-tet-tri | 5 | 0.004 |
| 11 | ery-gen-kan-lin-neo-oxa-pen-str-sul-tet-tri | 1 | 0.001 |
| 10 | aug-bac-enr-ery-gen-kan-oxa-pen-sul-tri | 1 | 0.001 |
| 10 | aug-cep-enr-ery-gen-kan-oxa-pen-sul-tet | 1 | 0.001 |
| 10 | aug-cep-enr-ery-kan-lin-neo-oxa-pen-sul | 1 | 0.001 |
| 10 | aug-cep-ery-kan-lin-neo-oxa-pen-sul-tet | 1 | 0.001 |
| 10 | aug-cep-ery-kan-lin-neo-oxa-pen-sul-tri | 1 | 0.001 |
| 10 | bac-ery-kan-lin-neo-pen-str-sul-tet-tri | 2 | 0.002 |
| 10 | cep-enr-gen-kan-lin-neo-nov-oxa-pen-sul | 1 | 0.001 |
| 10 | cep-enr-gen-kan-lin-neo-oxa-pen-sul-tri | 1 | 0.001 |
| 10 | cep-ery-gen-kan-lin-neo-oxa-pen-sul-tet | 1 | 0.001 |
| 10 | enr-ery-gen-kan-lin-neo-pen-sul-tet-tri | 5 | 0.004 |
| 10 | enr-ery-gen-kan-lin-oxa-pen-sul-tet-tri | 2 | 0.002 |
| 10 | enr-ery-kan-lin-neo-oxa-pen-sul-tet-tri | 1 | 0.001 |
| 10 | ery-gen-kan-lin-neo-oxa-pen-str-sul-tri | 1 | 0.001 |
| 10 | ery-gen-kan-lin-neo-oxa-pen-str-tet-tri | 1 | 0.001 |
| 10 | ery-gen-kan-lin-neo-pen-str-sul-tet-tri | 2 | 0.002 |
| 9 | aug-bac-cep-enr-ery-kan-neo-oxa-pen | 2 | 0.002 |
| 9 | aug-cep-enr-ery-kan-lin-neo-oxa-pen | 1 | 0.001 |
| 9 | aug-cep-gen-kan-lin-oxa-pen-sul-tri | 1 | 0.001 |
| 9 | aug-enr-ery-kan-lin-neo-oxa-pen-sul | 1 | 0.001 |
| 9 | aug-ery-gen-kan-oxa-pen-sul-tet-tri | 1 | 0.001 |
| 9 | bac-enr-ery-gen-kan-lin-neo-pen-tri | 1 | 0.001 |
| 9 | bac-ery-gen-kan-neo-pen-sul-tet-tri | 1 | 0.001 |
| 9 | bac-ery-kan-lin-neo-oxa-pen-sul-tri | 1 | 0.001 |
| 9 | bac-ery-kan-lin-neo-pen-str-sul-tri | 1 | 0.001 |
| 9 | bac-ery-kan-lin-neo-pen-sul-tet-tri | 4 | 0.004 |
| 9 | bac-ery-kan-neo-pen-str-sul-tet-tri | 1 | 0.001 |
| 9 | cep-enr-ery-kan-oxa-pen-sul-tet-tri | 1 | 0.001 |
| 9 | cep-ery-gen-kan-lin-neo-oxa-pen-sul | 1 | 0.001 |
| 9 | cep-gen-kan-lin-neo-oxa-pen-sul-tet | 1 | 0.001 |
| 9 | enr-ery-gen-kan-lin-neo-oxa-pen-tet | 1 | 0.001 |
| 9 | enr-ery-gen-kan-lin-neo-pen-sul-tri | 2 | 0.002 |
| 9 | enr-ery-kan-lin-neo-oxa-pen-sul-tet | 1 | 0.001 |
| 9 | enr-ery-kan-lin-neo-oxa-sul-tet-tri | 1 | 0.001 |
| 9 | enr-ery-kan-lin-neo-pen-sul-tet-tri | 4 | 0.004 |
| 9 | enr-gen-kan-neo-oxa-pen-sul-tet-tri | 1 | 0.001 |
| 9 | ery-gen-kan-lin-neo-oxa-pen-sul-tet | 1 | 0.001 |
| 9 | ery-gen-kan-lin-neo-oxa-pen-sul-tri | 2 | 0.002 |
| 9 | ery-gen-kan-lin-neo-oxa-pen-tet-tri | 1 | 0.001 |
| 9 | ery-gen-kan-lin-neo-pen-str-sul-tet | 1 | 0.001 |
| 9 | ery-gen-kan-lin-neo-pen-sul-tet-tri | 6 | 0.005 |
| 9 | ery-gen-kan-lin-pen-str-sul-tet-tri | 1 | 0.001 |
| 9 | ery-gen-kan-neo-pen-str-sul-tet-tri | 1 | 0.001 |
| 9 | ery-kan-lin-neo-pen-str-sul-tet-tri | 12 | 0.011 |
| 8 | aug-cep-enr-ery-kan-neo-oxa-sul | 1 | 0.001 |
| 8 | bac-enr-kan-neo-oxa-pen-sul-tet | 1 | 0.001 |
| 8 | bac-enr-kan-oxa-pen-sul-tet-tri | 1 | 0.001 |
| 8 | bac-ery-gen-kan-lin-oxa-pen-sul | 1 | 0.001 |
| 8 | bac-ery-gen-kan-lin-pen-sul-tet | 1 | 0.001 |
| 8 | bac-ery-gen-kan-neo-oxa-pen-sul | 2 | 0.002 |
| 8 | bac-ery-kan-lin-neo-oxa-pen-sul | 1 | 0.001 |
| 8 | bac-ery-kan-lin-neo-pen-str-sul | 2 | 0.002 |
| 8 | bac-ery-kan-lin-neo-pen-sul-tet | 1 | 0.001 |
| 8 | bac-ery-kan-lin-pen-str-sul-tet | 3 | 0.003 |
| 8 | bac-kan-neo-pen-str-sul-tet-tri | 1 | 0.001 |
| 8 | cep-enr-ery-kan-lin-neo-oxa-pen | 1 | 0.001 |
| 8 | cep-gen-kan-oxa-pen-sul-tet-tri | 1 | 0.001 |
| 8 | enr-ery-gen-kan-lin-oxa-pen-tet | 1 | 0.001 |
| 8 | enr-ery-gen-kan-neo-oxa-pen-tet | 1 | 0.001 |
| 8 | ery-gen-kan-lin-neo-oxa-pen-sul | 1 | 0.001 |
| 8 | ery-gen-kan-lin-neo-oxa-pen-tri | 1 | 0.001 |
| 8 | ery-gen-kan-lin-neo-pen-sul-tet | 2 | 0.002 |
| 8 | ery-gen-kan-lin-neo-pen-sul-tri | 1 | 0.001 |
| 8 | ery-gen-kan-lin-pen-str-sul-tet | 1 | 0.001 |
| 8 | ery-gen-kan-lin-pen-str-sul-tri | 1 | 0.001 |
| 8 | ery-kan-lin-neo-oxa-pen-sul-tet | 1 | 0.001 |
| 8 | ery-kan-lin-neo-pen-str-sul-tet | 15 | 0.013 |
| 8 | ery-kan-lin-neo-pen-str-sul-tri | 5 | 0.004 |
| 8 | ery-kan-lin-neo-pen-sul-tet-tri | 19 | 0.017 |
| 8 | ery-kan-lin-oxa-pen-str-sul-tri | 1 | 0.001 |
| 8 | ery-kan-lin-oxa-pen-sul-tet-tri | 1 | 0.001 |
| 8 | ery-kan-lin-pen-str-sul-tet-tri | 10 | 0.009 |
| 8 | ery-kan-neo-pen-str-sul-tet-tri | 1 | 0.001 |
| 8 | gen-kan-neo-pen-str-sul-tet-tri | 1 | 0.001 |
| 7 | aug-bac-cep-lin-nov-oxa-pen | 1 | 0.001 |
| 7 | bac-enr-ery-kan-neo-pen-tet | 1 | 0.001 |
| 7 | bac-gen-kan-lin-oxa-pen-tri | 1 | 0.001 |
| 7 | bac-kan-lin-neo-nov-oxa-str | 1 | 0.001 |
| 7 | cep-enr-gen-kan-oxa-pen-sul | 1 | 0.001 |
| 7 | enr-ery-kan-lin-oxa-pen-sul | 1 | 0.001 |
| 7 | enr-ery-oxa-pen-sul-tet-tri | 1 | 0.001 |
| 7 | ery-gen-kan-lin-neo-pen-sul | 4 | 0.004 |
| 7 | ery-gen-kan-lin-pen-str-sul | 1 | 0.001 |
| 7 | ery-gen-kan-lin-pen-sul-tri | 1 | 0.001 |
| 7 | ery-gen-kan-neo-oxa-pen-sul | 1 | 0.001 |
| 7 | ery-gen-kan-neo-pen-sul-tri | 1 | 0.001 |
| 7 | ery-kan-lin-neo-pen-str-sul | 8 | 0.007 |
| 7 | ery-kan-lin-neo-pen-sul-tet | 38 | 0.034 |
| 7 | ery-kan-lin-neo-pen-sul-tri | 11 | 0.010 |
| 7 | ery-kan-lin-neo-pen-tet-tri | 1 | 0.001 |
| 7 | ery-kan-lin-nov-pen-sul-tri | 1 | 0.001 |
| 7 | ery-kan-lin-pen-str-sul-tet | 17 | 0.015 |
| 7 | ery-kan-lin-pen-str-sul-tri | 1 | 0.001 |
| 7 | ery-kan-lin-pen-sul-tet-tri | 5 | 0.004 |
| 7 | ery-kan-neo-pen-str-sul-tet | 3 | 0.003 |
| 7 | ery-kan-neo-pen-sul-tet-tri | 2 | 0.002 |
| 7 | ery-kan-pen-str-sul-tet-tri | 2 | 0.002 |
| 7 | ery-lin-neo-sul-tet-tri | 1 | 0.001 |
| 7 | gen-kan-neo-pen-sul-tet-tri | 2 | 0.002 |
| 7 | gen-kan-oxa-pen-sul-tet-tri | 2 | 0.002 |
| 7 | gen-kan-pen-str-sul-tet-tri | 1 | 0.001 |
| 7 | kan-lin-neo-pen-sul-tet-tri | 1 | 0.001 |
| 6 | aug-bac-cep-oxa-pen-sul | 1 | 0.001 |
| 6 | aug-cep-enr-oxa-pen-tet | 1 | 0.001 |
| 6 | aug-cep-oxa-pen-sul-tet | 1 | 0.001 |
| 6 | aug-enr-ery-kan-neo-pen | 1 | 0.001 |
| 6 | aug-ery-oxa-pen-sul-tet | 1 | 0.001 |
| 6 | bac-enr-oxa-pen-sul-tri | 1 | 0.001 |
| 6 | bac-ery-kan-neo-oxa-sul | 1 | 0.001 |
| 6 | bac-ery-lin-oxa-pen-tet | 1 | 0.001 |
| 6 | bac-kan-pen-sul-tet-tri | 1 | 0.001 |
| 6 | bac-lin-pen-sul-tet-tri | 1 | 0.001 |
| 6 | bac-oxa-pen-sul-tet-tri | 2 | 0.002 |
| 6 | cep-enr-oxa-pen-tet-tri | 1 | 0.001 |
| 6 | cep-ery-lin-oxa-pen-tri | 1 | 0.001 |
| 6 | cep-gen-kan-oxa-pen-sul | 1 | 0.001 |
| 6 | ery-gen-kan-lin-pen-sul | 1 | 0.001 |
| 6 | ery-gen-kan-neo-pen-sul | 1 | 0.001 |
| 6 | ery-kan-lin-neo-pen-sul | 21 | 0.019 |
| 6 | ery-kan-lin-neo-pen-tet | 3 | 0.003 |
| 6 | ery-kan-lin-neo-sul-tet | 1 | 0.001 |
| 6 | ery-kan-lin-pen-str-sul | 4 | 0.004 |
| 6 | ery-kan-lin-pen-str-tet | 1 | 0.001 |
| 6 | ery-kan-lin-pen-sul-tet | 19 | 0.017 |
| 6 | ery-kan-lin-pen-sul-tri | 1 | 0.001 |
| 6 | ery-kan-lin-sul-tet-tri | 1 | 0.001 |
| 6 | ery-kan-neo-oxa-pen-sul | 1 | 0.001 |
| 6 | ery-kan-neo-pen-str-sul | 2 | 0.002 |
| 6 | ery-kan-neo-pen-sul-tet | 6 | 0.005 |
| 6 | ery-kan-neo-pen-sul-tri | 1 | 0.001 |
| 6 | ery-kan-oxa-pen-sul-tri | 1 | 0.001 |
| 6 | ery-kan-pen-str-sul-tet | 3 | 0.003 |
| 6 | ery-lin-oxa-pen-sul-tri | 1 | 0.001 |
| 6 | ery-lin-pen-str-sul-tet | 1 | 0.001 |
| 6 | ery-pen-str-sul-tet-tri | 1 | 0.001 |
| 6 | gen-kan-neo-pen-sul-tri | 1 | 0.001 |
| 6 | gen-kan-oxa-pen-sul-tri | 1 | 0.001 |
| 6 | gen-kan-pen-str-sul-tri | 1 | 0.001 |
| 6 | gen-kan-pen-sul-tet-tri | 14 | 0.012 |
| 6 | kan-neo-pen-sul-tet-tri | 1 | 0.001 |
| 6 | kan-oxa-pen-str-sul-tet | 1 | 0.001 |
| 6 | kan-oxa-pen-sul-tet-tri | 2 | 0.002 |
| 6 | kan-pen-str-sul-tet-tri | 2 | 0.002 |
| 6 | oxa-pen-str-sul-tet-tri | 1 | 0.001 |
| 5 | aug-bac-nov-oxa-pen | 1 | 0.001 |
| 5 | aug-bac-oxa-pen-tet | 1 | 0.001 |
| 5 | aug-cep-enr-oxa-pen | 2 | 0.002 |
| 5 | bac-ery-kan-neo-pen | 1 | 0.001 |
| 5 | bac-gen-kan-neo-sul | 1 | 0.001 |
| 5 | bac-gen-kan-pen-sul | 1 | 0.001 |
| 5 | bac-kan-lin-oxa-str | 1 | 0.001 |
| 5 | bac-kan-neo-pen-tet | 1 | 0.001 |
| 5 | bac-nov-oxa-pen-str | 1 | 0.001 |
| 5 | bac-oxa-pen-sul-tet | 1 | 0.001 |
| 5 | bac-oxa-pen-sul-tri | 1 | 0.001 |
| 5 | bac-pen-str-sul-tet | 2 | 0.002 |
| 5 | bac-pen-str-sul-tri | 1 | 0.001 |
| 5 | bac-pen-sul-tet-tri | 11 | 0.010 |
| 5 | cep-lin-nov-oxa-pen | 1 | 0.001 |
| 5 | enr-pen-sul-tet-tri | 1 | 0.001 |
| 5 | ery-kan-lin-neo-pen | 4 | 0.004 |
| 5 | ery-kan-lin-neo-sul | 1 | 0.001 |
| 5 | ery-kan-lin-neo-tri | 1 | 0.001 |
| 5 | ery-kan-lin-pen-str | 1 | 0.001 |
| 5 | ery-kan-lin-pen-sul | 7 | 0.006 |
| 5 | ery-kan-lin-pen-tet | 4 | 0.004 |
| 5 | ery-kan-lin-str-sul | 1 | 0.001 |
| 5 | ery-kan-neo-pen-sul | 2 | 0.002 |
| 5 | ery-kan-neo-pen-tet | 1 | 0.001 |
| 5 | ery-kan-pen-str-sul | 1 | 0.001 |
| 5 | ery-kan-pen-sul-tet | 8 | 0.007 |
| 5 | ery-lin-pen-sul-tet | 5 | 0.004 |
| 5 | ery-lin-pen-sul-tri | 1 | 0.001 |
| 5 | ery-oxa-pen-sul-tet | 2 | 0.002 |
| 5 | gen-kan-oxa-pen-sul | 1 | 0.001 |
| 5 | gen-kan-oxa-pen-tri | 1 | 0.001 |
| 5 | gen-kan-pen-str-tet | 1 | 0.001 |
| 5 | gen-kan-pen-sul-tet | 5 | 0.004 |
| 5 | gen-kan-pen-sul-tri | 9 | 0.008 |
| 5 | kan-neo-pen-str-sul | 1 | 0.001 |
| 5 | kan-neo-pen-sul-tet | 2 | 0.002 |
| 5 | kan-neo-pen-sul-tri | 1 | 0.001 |
| 5 | kan-nov-str-sul-tet | 2 | 0.002 |
| 5 | kan-pen-str-sul-tet | 1 | 0.001 |
| 5 | kan-pen-sul-tet-tri | 7 | 0.006 |
| 5 | lin-oxa-pen-str-sul | 2 | 0.002 |
| 5 | lin-pen-sul-tet-tri | 1 | 0.001 |
| 5 | oxa-pen-sul-tet-tri | 4 | 0.004 |
| 4 | aug-cep-oxa-pen | 1 | 0.001 |
| 4 | aug-enr-ery-pen | 1 | 0.001 |
| 4 | aug-ery-oxa-pen | 1 | 0.001 |
| 4 | bac-ery-lin-pen | 1 | 0.001 |
| 4 | bac-ery-nov-pen | 1 | 0.001 |
| 4 | bac-kan-neo-pen | 1 | 0.001 |
| 4 | bac-nov-oxa-pen | 1 | 0.001 |
| 4 | bac-nov-pen-sul | 1 | 0.001 |
| 4 | bac-oxa-pen-tet | 1 | 0.001 |
| 4 | bac-pen-sul-tet | 5 | 0.004 |
| 4 | bac-pen-sul-tri | 9 | 0.008 |
| 4 | cep-enr-oxa-pen | 1 | 0.001 |
| 4 | cep-ery-nov-pen | 1 | 0.001 |
| 4 | cep-nov-oxa-pen | 1 | 0.001 |
| 4 | cep-oxa-pen-sul | 1 | 0.001 |
| 4 | enr-oxa-pen-sul | 2 | 0.002 |
| 4 | enr-pen-sul-tet | 1 | 0.001 |
| 4 | enr-pen-sul-tri | 3 | 0.003 |
| 4 | ery-gen-kan-pen | 1 | 0.001 |
| 4 | ery-kan-lin-sul | 1 | 0.001 |
| 4 | ery-kan-pen-str | 1 | 0.001 |
| 4 | ery-kan-pen-sul | 3 | 0.003 |
| 4 | ery-kan-sul-tet | 1 | 0.001 |
| 4 | ery-lin-pen-str | 1 | 0.001 |
| 4 | ery-lin-pen-sul | 1 | 0.001 |
| 4 | ery-lin-pen-tet | 1 | 0.001 |
| 4 | ery-oxa-pen-sul | 3 | 0.003 |
| 4 | ery-pen-sul-tet | 2 | 0.002 |
| 4 | ery-pen-sul-tri | 4 | 0.004 |
| 4 | gen-kan-oxa-sul | 1 | 0.001 |
| 4 | gen-kan-pen-sul | 4 | 0.004 |
| 4 | gen-kan-pen-tet | 1 | 0.001 |
| 4 | gen-kan-sul-tet | 1 | 0.001 |
| 4 | gen-kan-sul-tri | 1 | 0.001 |
| 4 | kan-neo-pen-sul | 2 | 0.002 |
| 4 | kan-neo-sul-tet | 1 | 0.001 |
| 4 | kan-neo-sul-tri | 1 | 0.001 |
| 4 | kan-nov-pen-sul | 1 | 0.001 |
| 4 | kan-oxa-sul-tri | 1 | 0.001 |
| 4 | kan-pen-sul-tet | 4 | 0.004 |
| 4 | kan-pen-sul-tri | 3 | 0.003 |
| 4 | lin-nov-pen-tet | 1 | 0.001 |
| 4 | lin-oxa-pen-sul | 1 | 0.001 |
| 4 | lin-pen-sul-tet | 1 | 0.001 |
| 4 | nov-oxa-pen-tet | 1 | 0.001 |
| 4 | nov-pen-sul-tri | 1 | 0.001 |
| 4 | oxa-pen-sul-tet | 3 | 0.003 |
| 4 | oxa-str-sul-tri | 1 | 0.001 |
| 4 | pen-str-sul-tet | 1 | 0.001 |
| 4 | pen-str-sul-tri | 10 | 0.009 |
| 4 | pen-sul-tet-tri | 216 | 0.191 |
| 3 | aug-ery-pen | 1 | 0.001 |
| 3 | aug-pen-sul | 1 | 0.001 |
| 3 | bac-ery-lin | 1 | 0.001 |
| 3 | bac-ery-pen | 1 | 0.001 |
| 3 | bac-ery-tri | 1 | 0.001 |
| 3 | bac-lin-nov | 1 | 0.001 |
| 3 | bac-lin-pen | 1 | 0.001 |
| 3 | bac-nov-oxa | 4 | 0.004 |
| 3 | bac-oxa-pen | 2 | 0.002 |
| 3 | bac-pen-sul | 10 | 0.009 |
| 3 | bac-pen-tet | 1 | 0.001 |
| 3 | bac-pen-tri | 1 | 0.001 |
| 3 | bac-sul-tet | 1 | 0.001 |
| 3 | bac-sul-tri | 4 | 0.004 |
| 3 | cep-enr-oxa | 1 | 0.001 |
| 3 | cep-oxa-pen | 1 | 0.001 |
| 3 | cep-pen-sul | 1 | 0.001 |
| 3 | enr-ery-pen | 1 | 0.001 |
| 3 | enr-pen-tet | 1 | 0.001 |
| 3 | ery-kan-pen | 1 | 0.001 |
| 3 | ery-lin-pen | 2 | 0.002 |
| 3 | ery-lin-sul | 1 | 0.001 |
| 3 | ery-lin-tet | 2 | 0.002 |
| 3 | ery-pen-sul | 13 | 0.011 |
| 3 | ery-pen-tet | 4 | 0.004 |
| 3 | ery-sul-tet-tri | 2 | 0.002 |
| 3 | gen-kan-neo | 1 | 0.001 |
| 3 | gen-kan-pen | 4 | 0.004 |
| 3 | gen-kan-sul | 2 | 0.002 |
| 3 | gen-neo-oxa | 1 | 0.001 |
| 3 | kan-neo-pen | 1 | 0.001 |
| 3 | kan-oxa-sul | 1 | 0.001 |
| 3 | kan-pen-sul | 5 | 0.004 |
| 3 | kan-pen-tet | 1 | 0.001 |
| 3 | lin-nov-oxa | 1 | 0.001 |
| 3 | lin-nov-pen | 2 | 0.002 |
| 3 | lin-nov-sul | 2 | 0.002 |
| 3 | lin-oxa-pen | 3 | 0.003 |
| 3 | lin-oxa-sul | 1 | 0.001 |
| 3 | lin-pen-str | 1 | 0.001 |
| 3 | lin-pen-sul | 1 | 0.001 |
| 3 | lin-pen-tet | 1 | 0.001 |
| 3 | lin-sul-tet | 1 | 0.001 |
| 3 | lin-sul-tri | 1 | 0.001 |
| 3 | neo-pen-sul | 1 | 0.001 |
| 3 | nov-oxa-pen | 2 | 0.002 |
| 3 | nov-oxa-tet | 1 | 0.001 |
| 3 | nov-pen-sul | 1 | 0.001 |
| 3 | nov-pen-tet | 10 | 0.009 |
| 3 | nov-sul-tri | 1 | 0.001 |
| 3 | oxa-pen-str | 1 | 0.001 |
| 3 | oxa-pen-sul | 4 | 0.004 |
| 3 | oxa-pen-tet | 1 | 0.001 |
| 3 | oxa-sul-tet | 1 | 0.001 |
| 3 | oxa-sul-tri | 2 | 0.002 |
| 3 | pen-str-sul | 12 | 0.011 |
| 3 | pen-str-tet | 1 | 0.001 |
| 3 | pen-sul-tet | 369 | 0.326 |
| 3 | pen-sul-tri | 168 | 0.148 |
| 3 | pen-tet-tri | 1 | 0.001 |
| 3 | sul-tet-tri | 11 | 0.010 |
| 2 | aug-pen | 5 | 0.004 |
| 2 | bac-lin | 1 | 0.001 |
| 2 | bac-nov | 7 | 0.006 |
| 2 | bac-pen | 14 | 0.012 |
| 2 | bac-sul | 25 | 0.022 |
| 2 | bac-tet | 2 | 0.002 |
| 2 | cep-oxa | 1 | 0.001 |
| 2 | cep-pen | 1 | 0.001 |
| 2 | enr-ery | 1 | 0.001 |
| 2 | enr-oxa | 2 | 0.002 |
| 2 | enr-pen | 1 | 0.001 |
| 2 | enr-sul | 2 | 0.002 |
| 2 | ery-lin | 2 | 0.002 |
| 2 | ery-pen | 11 | 0.010 |
| 2 | ery-sul | 3 | 0.003 |
| 2 | ery-tet | 2 | 0.002 |
| 2 | gen-kan | 9 | 0.008 |
| 2 | kan-pen | 2 | 0.002 |
| 2 | kan-sul | 2 | 0.002 |
| 2 | kan-tri | 1 | 0.001 |
| 2 | lin-neo | 1 | 0.001 |
| 2 | lin-nov | 5 | 0.004 |
| 2 | lin-oxa | 3 | 0.003 |
| 2 | lin-pen | 1 | 0.001 |
| 2 | lin-sul | 3 | 0.003 |
| 2 | lin-tet | 1 | 0.001 |
| 2 | neo-pen | 1 | 0.001 |
| 2 | nov-oxa | 1 | 0.001 |
| 2 | nov-pen | 20 | 0.018 |
| 2 | nov-sul | 1 | 0.001 |
| 2 | nov-tet | 5 | 0.004 |
| 2 | oxa-pen | 5 | 0.004 |
| 2 | oxa-str | 2 | 0.002 |
| 2 | oxa-tri | 5 | 0.004 |
| 2 | pen-str | 3 | 0.003 |
| 2 | pen-sul | 617 | 0.545 |
| 2 | pen-tet | 172 | 0.152 |
| 2 | pen-tri | 7 | 0.006 |
| 2 | str-sul | 4 | 0.004 |
| 2 | str-tet | 1 | 0.001 |
| 2 | sul-tet | 27 | 0.024 |
| 2 | sul-tri | 51 | 0.045 |
| 1 | bac | 24 | 0.021 |
| 1 | enr | 9 | 0.008 |
| 1 | ery | 8 | 0.007 |
| 1 | kan | 10 | 0.009 |
| 1 | lin | 7 | 0.006 |
| 1 | nov | 21 | 0.019 |
| 1 | oxa | 11 | 0.010 |
| 1 | pen | 632 | 0.558 |
| 1 | str | 4 | 0.004 |
| 1 | sul | 544 | 0.481 |
| 1 | tet | 37 | 0.033 |
| 1 | tri | 5 | 0.004 |
|  | Total | 1132 | 100 |

^1^UKVDL= University of Kentucky Veterinary Diagnostic Laboratory

^2^aug = amoxicillin-clavulanic

^3^bac = bacitracin

^4^cep = cephalothin

^5^enr = enrofloxacin

^6^ery = erythromycin

^7^gen = gentamicin

^8^kan = kanamycin

^9^lin = lincomycin

^10^neo = neomycin

^11^nov = novobiocin

^12^oxa = oxcacillin

^13^pen = penicillin

^14^str = streptomycin

^15^sul = sulfadiazine-trimethoprim

^16^tet = tetracycline

^17^tri = sulfamethoxazole

Freq = frequency

Abx # = Number of antibiotics isolates are resistant to
